# On the Metal-Aided Catalytic Mechanism for Phosphodiester Bond Cleavage Performed by Nanozymes

**DOI:** 10.1021/acscatal.1c01215

**Published:** 2021-07-02

**Authors:** Adam Pecina, Daniele Rosa-Gastaldo, Laura Riccardi, Sebastian Franco-Ulloa, Emil Milan, Paolo Scrimin, Fabrizio Mancin, Marco De Vivo

**Affiliations:** † Laboratory of Molecular Modeling and Drug Discovery, Istituto Italiano di Tecnologia, Via Morego 30, 16163 Genoa, Italy; ‡ Dipartimento di Scienze Chimiche, Università di Padova, Via Marzolo 1, 35131 Padova, Italy

**Keywords:** functionalized nanoparticles, artificial enzymes, monolayer-protected gold clusters, metal-dependent catalysis, molecular simulations, MD, NMR, nanotechnology

## Abstract

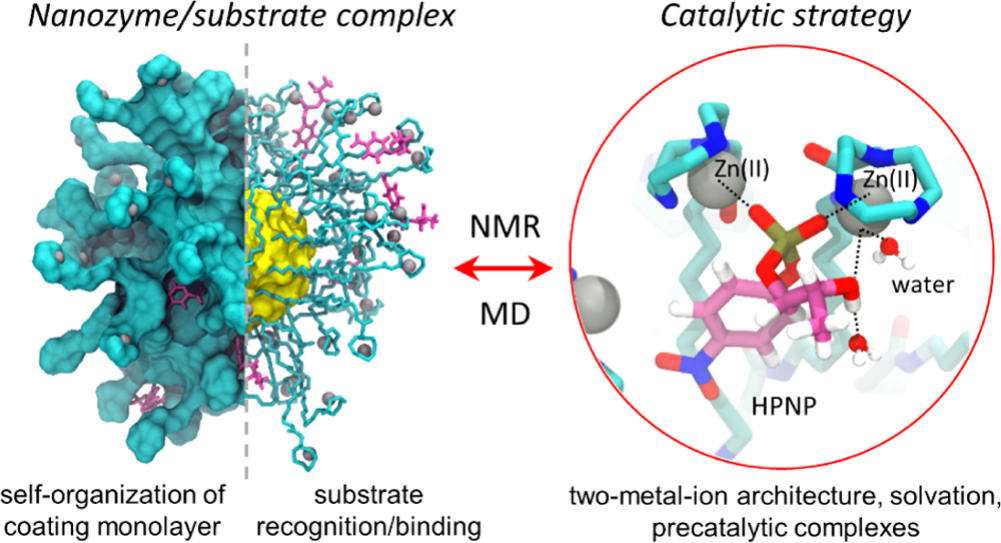

Recent studies have shown that gold nanoparticles (AuNPs) functionalized with Zn(II) complexes can cleave phosphate esters and nucleic acids. Remarkably, such synthetic nanonucleases appear to catalyze metal (Zn)-aided hydrolytic reactions of nucleic acids similar to metallonuclease enzymes. To clarify the reaction mechanism of these nanocatalysts, here we have comparatively analyzed two nanonucleases with a >10-fold difference in the catalytic efficiency for the hydrolysis of the 2-hydroxypropyl-4-nitrophenylphosphate (HPNP, a typical RNA model substrate). We have used microsecond-long atomistic simulations, integrated with NMR experiments, to investigate the structure and dynamics of the outer coating monolayer of these nanoparticles, either alone or in complex with HPNP, in solution. We show that the most efficient one is characterized by coating ligands that promote a well-organized monolayer structure, with the formation of solvated bimetallic catalytic sites. Importantly, we have found that these nanoparticles can mimic two-metal-ion enzymes for nucleic acid processing, with Zn ions that promote HPNP binding at the reaction center. Thus, the two-metal-ion-aided hydrolytic strategy of such nanonucleases helps in explaining their catalytic efficiency for substrate hydrolysis, in accordance with the experimental evidence. These mechanistic insights reinforce the parallelism between such functionalized AuNPs and proteins toward the rational design of more efficient catalysts.

## Introduction

Nanoparticles with catalytic abilities have attracted considerable interest over the last few years, earning the definition of “nanozymes”.^1^ Even if used in very different contexts,^2^ this name was introduced by Scrimin in 2004 to indicate catalytic nanoparticles whose structural and mechanistic features mimic those of enzymes.^3^ In this regard, monolayer-protected gold nanoparticles (AuNPs) have unique self-organization and molecular recognition properties that are exploitable in a wide range of applications, for example, pollutant removal, chemosensing, cancer theragnostics, and also catalysis.^4^ Such properties arise exactly from the outer organic monolayer that can be differently functionalized in order to perform specific cooperative functions, providing reactive groups and stabilizing interactions for carboxylic ester cleavage, asymmetric dihydroxylation reactions, electrocatalytic reductions, cycloadditions, and even the hydrolysis of phosphodiester bonds in nucleic acids.^5^

The latter reaction, in particular, is a fundamental biochemical process often accelerated by enzymes featuring divalent metal ions located at the reaction center. In selected cases, for example, in nucleic-acid-processing enzymes, more than one metal ion is known to be essential, and the substrate hydrolysis occurs via the recognized two-metal-ion mechanism.^6^ Through such a catalytic mechanism, the two ions help in the activation of the nucleophile and in the stabilization of the transition state, eventually favoring also the departure of the leaving group. In analogy, artificial catalytic systems have been developed in order to promote metal-aided phosphatase activity, with Zn(II) and Cu(II) complexes as the most efficient metals for multinuclear catalysts.^7^ Remarkably, even faster accelerations have been achieved by polymeric and supramolecular assemblies containing multiple Zn(II) chelating centers.^3a,8^ To this aim, small gold clusters (about 2 nm) passivated by a monolayer of organic ligands or short peptides have been engineered to recreate self-organized catalytic sites with properties similar to those of phosphate-cleaving enzymes,^4b,9^ resulting in highly efficient artificial “nanonucleases”.^4b,5f,10^

**Figure 1:**
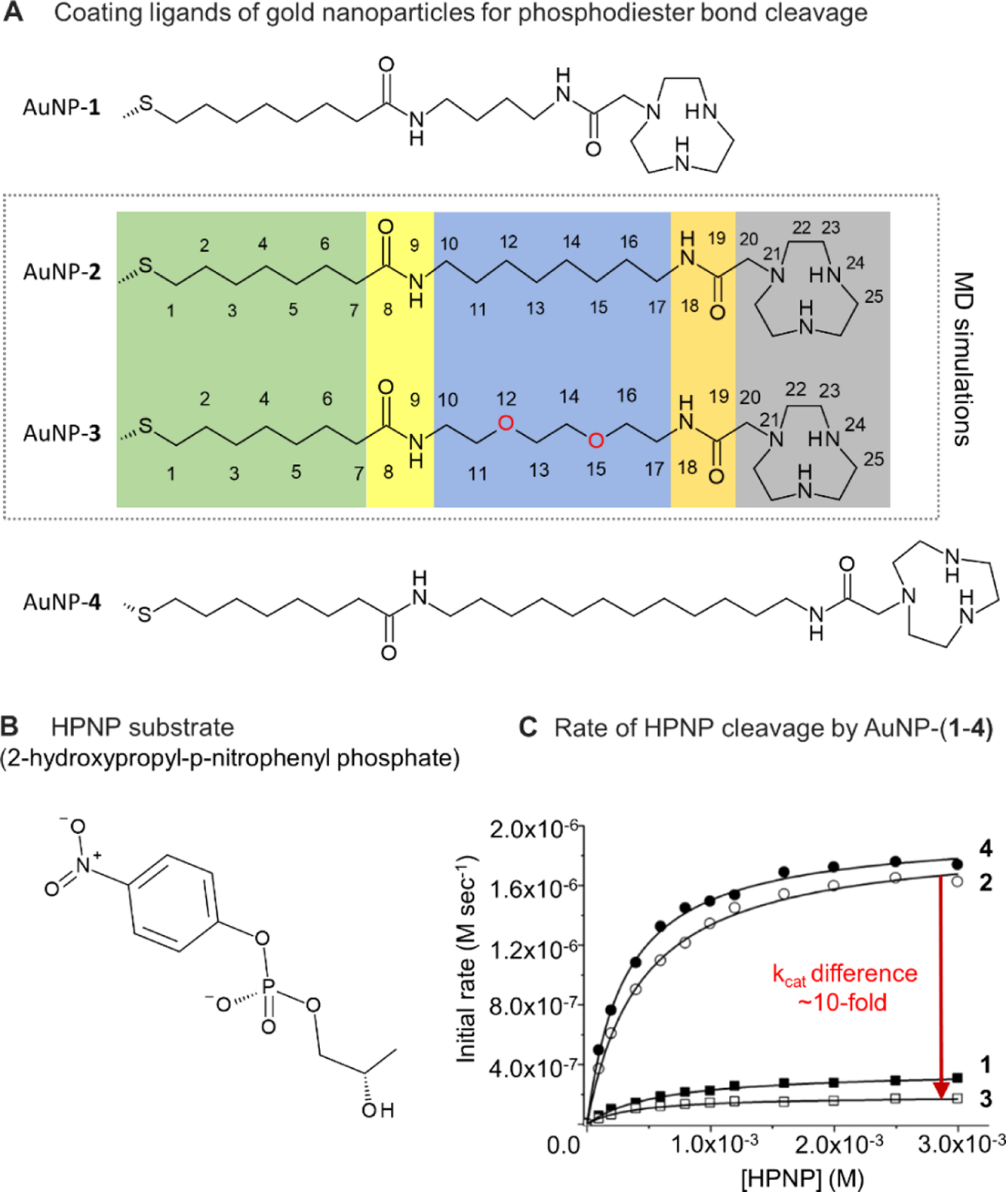
(A) Coating ligands of four phosphodiester bond-cleaving nanozymes from ref (10a). The individual components of the coating ligands of AuNP-**2** and AuNP-**3**, two nanoparticles (similar in structure and different in activity) selected for this study, are divided into 5 main segments: (i) the region most proximal to the gold core, which is a hydrophobic C7 alkyl chain (in green); (ii) an inner amide group (in yellow); (iii) a linker chain that differs in the two nanoparticles, being a hydrophobic alkyl chain in AuNP-**2** and a hydrophilic polyethylenglycol (PEG) in AuNP-**3** (in blue); (iv) an outer amide group (in orange); and (v) the terminal TACN crown which chelates Zn ions (in gray). (B) Chemical structure of the HPNP substrate. (C) Data from ref (10a), on the dependence of the initial rate of HPNP at fixed AuNP-**2**(**3**)/Zn concentration as a function of the substrate HPNP concentration, with an activity drop (∼10-fold, highlighted in red) between AuNP-**2** and AuNP-**3**. Specifically, *k*_cat_ = 0.21 s^–1^ for AuNP-**2** and *k*_cat_ = 0.02 s^–1^ for AuNP-**3** for the hydrolysis of HPNP, while *K*_M_ is 0.40 nM for AuNP-**2** and 0.38 nM for AuNP-**3**.^10a^

The cleavage of the phosphodiester bond by such nanozymes was first demonstrated using an RNA model substrate, that is, 2-hydroxypropyl-4-nitrophenylphosphate (HPNP), whose transesterification was accelerated by nanonucleases featuring a 2 nm diameter gold core coated with a monolayer of thiols bearing a 1,4,7-triazacyclononane (TACN) moiety.^10a,3a^ Rate accelerations with these nanoparticles reached ∼7 orders of magnitude with respect to the background reaction, and Michaelis–Menten reactivity profiles were observed.^10a,11^ Interestingly, studies showed that the chemical structure of coating thiols can influence significantly the nanoparticle’s reactivity (Figure 1). In particular, the elongation of the alkyl spacer unit of AuNP-**1**, connecting the TACN unit with the nanoparticle surface, from 4 to 8 and 12 carbon atoms led to a 5-fold better reactivity (*k*_cat_) in AuNP-**2** and **4**. However, the simple substitution of the alkyl spacer of AuNP-**2** with a oligo(oxyethylene) moiety, as in AuNP-**3**, caused an ∼10-fold drop in *k*_cat_. Precisely, *k*_cat_ = 0.21 s^–1^ for AuNP-**2** and *k*_cat_ = 0.02 s^–1^ for AuNP-**3**.^10a^ However, intriguingly, the substrate affinity (*K*_M_) was similar in all such AuNPs, ruling out the possibility of differential hydrophobic contributions to binding and product release.^10a^ The most credited mechanistic hypothesis to explain such different *k*_cat_ between AuNPs with highly similar structures and *K*_M_ is the possible distinctive solvation at their reaction center, where the decreased polarity of the alkyl monolayer of AuNP-**2** with respect to AuNP-**3** would increase the catalytic effectiveness.^10a^

To examine such a hypothesis and dissect the mechanistic origin of the catalytic power of these nanoparticles, here we have performed a comparative study of AuNP-**2** and AuNP-**3**, which feature a >10-fold difference in the catalytic efficiency in spite of their elevated structural similarities and similar substrate affinity (Figure 1).^10a^ To this regard, we have recently used molecular dynamics (MD) simulations to show that functionalized nanoparticles can form binding pockets in the coating monolayer, resembling the active sites of natural enzymes.^10b,12^ We have also shown that nanonucleases can indeed form precatalytic complexes with DNA, in solution.^10b^ In this context, through the use of microsecond-long atomistic simulations integrated with NMR experiments, here we have studied the specific structure and dynamics of the outer coating monolayer of such catalytic nanoparticles, with the ultimate goal to better understand their catalytic strategy for substrate hydrolysis.

## Results and Discussion

### Thiols in AuNP-**2** Are More Extended than Those in AuNP-**3**

**Figure 2:**
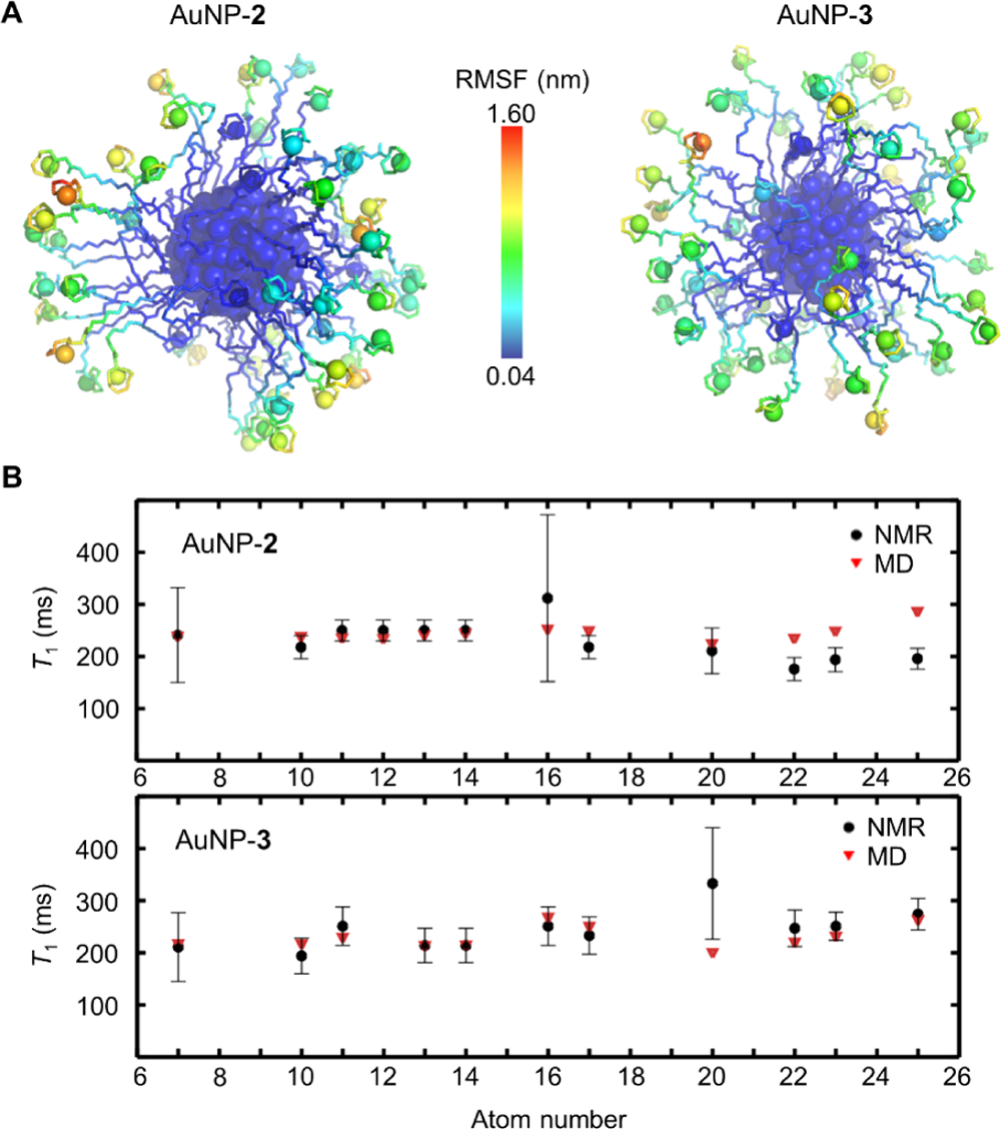
(A) Representative structures (at 100 ns) of AuNP-**2** and AuNP-**3** with a color scheme based on the RMSF of all heavy atoms, where the blue color indicates small fluctuations, that is, rigid atoms during the simulations, and the red color indicates high fluctuations, that is, highly flexible atoms. (B) Comparison of NMR-based and MD-derived *T*_1_ values of ^13^C nuclei along the chains of AuNP-**2** and AuNP-**3**. For a direct comparison, the MD simulations were performed in the absence of Zn ions to be in line with the NMR setup. Large errors in experimental data for atoms 7 and 16 for AuNP-**2** and for atoms 7 and 20 for AuNP-**3** are due to the low intensity of the relative signals.

The specific morphology of the coating monolayer in AuNP-**2** and AuNP-**3** is shaped by the (self)organization of the individual thiols on the surface of the gold core (Figures S1–S8). To investigate this point, we first performed NMR measurements of the *T*_1_ relaxation times for the ^13^C nuclei of AuNP-**2** and AuNP-**3**, featuring, respectively, core sizes of 1.6 ± 0.3 and 1.6 ± 0.4 nm (Figures S3 and S6). Relaxivities of the carbon nuclei depend indeed on the mobility of the coating ligands in the monolayer. Measurements were performed in the absence of Zn ions to allow a more reliable assignment of the signals (Figures S9–S12). In this way, we could clearly identify all the signals arising from carbons in the TACN crown and linker regions and from carbon 7 of the inner alkyl region. Results show a similar level of mobility for the outer portions of the ligands coating the two AuNPs. The narrow window of measured relaxivities (from 0.164 to 0.333 s) suggests also that there are small mobility differences along the chains (Figure 2 and Table S1).^12a^

These results were, here, integrated with extensive classical MD simulations of the two solvated nanoparticles (one 200 ns long MD simulations for each AuNP in the absence of Zn ions and four 200 ns long MD replicas for each AuNP in the presence of Zn ions; ∼2 μs simulation time in total). Notably, the Au_144_(SR)_60_ structure^13^ and model^14^ have been used in our calculations, as (i) they match well with the size (∼1.6 nm) of the main component of experimentally prepared nanoparticle batches with average sizes around 1.6–2.0 nm^15^ and (ii) they fit well to the properties of such nanoparticles, such as in the case of NMR-measured relaxation times.^12,16^

From these simulations, we first computed the root mean square fluctuations (RMSFs) and decays of rotational autocorrelation functions (RCFs) of the carbon atoms. This analysis confirms the increased degree of mobility, moving from the atoms closer to the gold core, which is more restrained, toward the more mobile terminal part of the thiol (Figures 2A and S13). Using these data, we also calculated the *T*_1_ relaxation times for the ^13^C nuclei via the Lipari–Szabo approach.^17^ These were then compared with the experimental results (reported in the paragraph above and shown in Figure 2B and Table S1). Accordingly, we found a good match between the calculated and measured relaxivities, confirming that the simulations correctly model the nanoparticles’ structure and dynamics (Figure 2B). Notably, this comparison also suggests that Zn ions—present in the MD simulations (and not in NMR experiments)—do not affect significantly the linker mobility (Figure S13 and Table S1).

**Figure 3:**
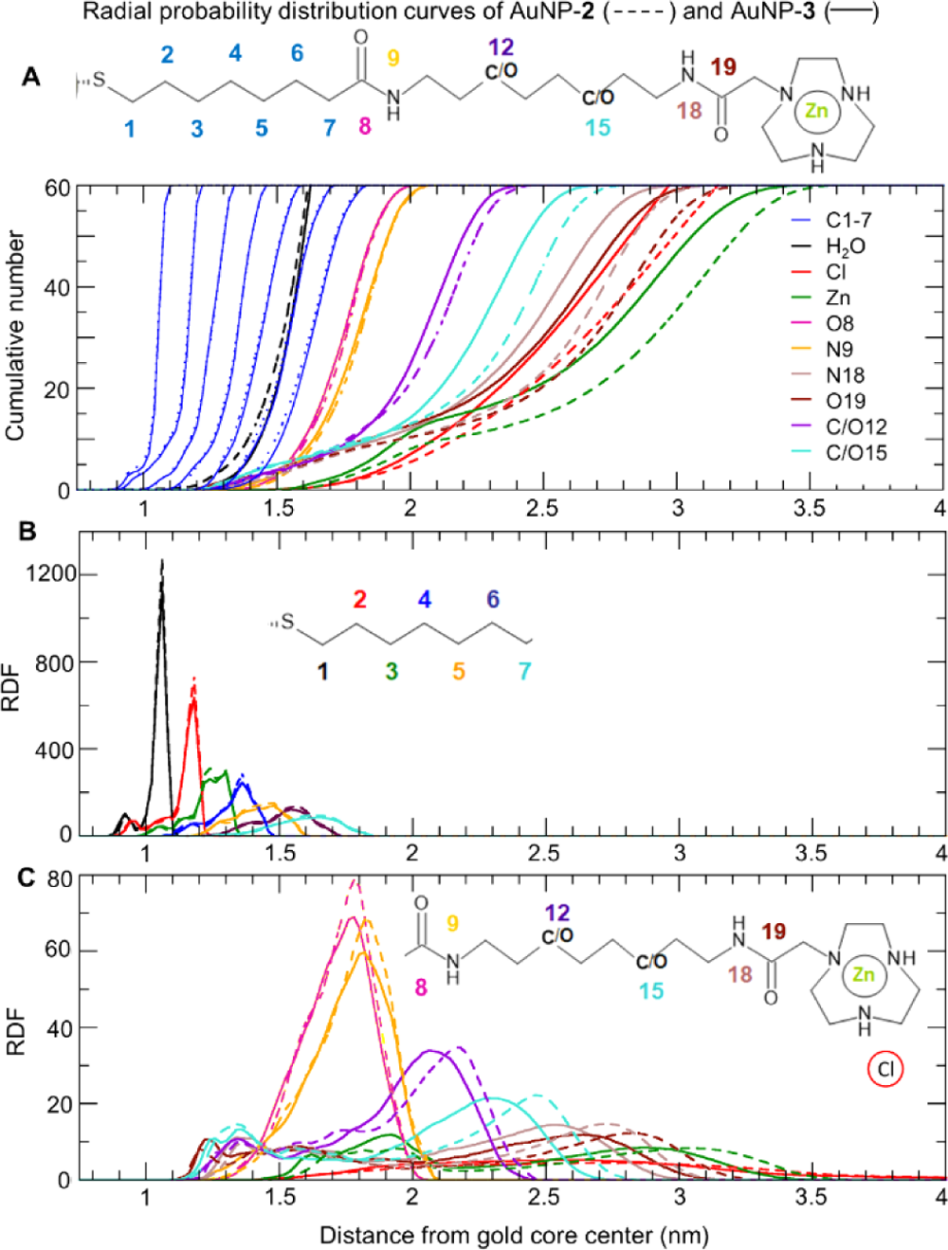
(A) Cumulative number RDFs of the selected atoms from both nanoparticles (AuNP-**2** in dashed lines and AuNP-**3** in solid lines) together with Cl ions and the oxygen atom of water molecules (H_2_O). (B) RDF functions of the first seven carbons of the hydrophobic alkyl chains of AuNP-**2** and AuNP-**3**. (C) RDF functions of the hydrophobic alkyl linker of AuNP-**2** and the hydrophilic PEG linker of AuNP-**3** together with Zn ions chelated by TACN crowns and Cl ions (in red).

Then, we analyzed the structural arrangement of the coating thiols around the gold core, examining inter- and intra-thiol interactions, and interactions with solvent molecules. First, the radial distribution functions (RDFs) of the selected atoms provide a description of the localization of each segment of the thiols on the nanoparticles’ surface (Figures 3 and S14). RDFs reveal that the inner hydrophobic alkyl chain and the inner amide region of the coating monolayer are similarly located in both AuNPs. In detail, the alkyl chains are identically packed and are found up to 1.8 nm from the gold core (Figure 3A,B). Distributions are very narrow in the proximity of the metal surface and become broader and broader by increasing the distance. This behavior well compares with the increasing mobility suggested by the RCF previously discussed. Also the distributions of the inner amide group (O8 and N9 atoms) are similar for both AuNPs, in between 1.2 and 2.1 nm, and quite broad (see Figure 3C).

On the contrary, the atoms of the linkers (represented by C12 and C15 in AuNP-**2** and O12 and O15 in AuNP-**3**) and the atoms of the outer amide group (N18 and O19 in both AuNPs) show broad and multipeak distributions with relevant differences between AuNP-**2** and AuNP-**3** (Figure 3A,C). In detail, RDFs of AuNP-**2** are shifted to greater distances compared to AuNP-**3**. The global maximum of C12 in AuNP-**2** is shifted by 0.1 nm when compared with the O12 atom, which is its equivalent in AuNP-**3**. The peaks of other atoms (C15, N18, O19, and Zn) are shifted by about 0.2 nm (Figure 3C). The RDFs thus suggest that the coating thiols of AuNP-**2** are more extended than those in AuNP-**3**. This different structural organization is also reflected in the radius of gyration, with values of 1.73 ± 0.02 nm for AuNP-**2** and 1.65 ± 0.01 nm for AuNP-**3**.

**Figure 4:**
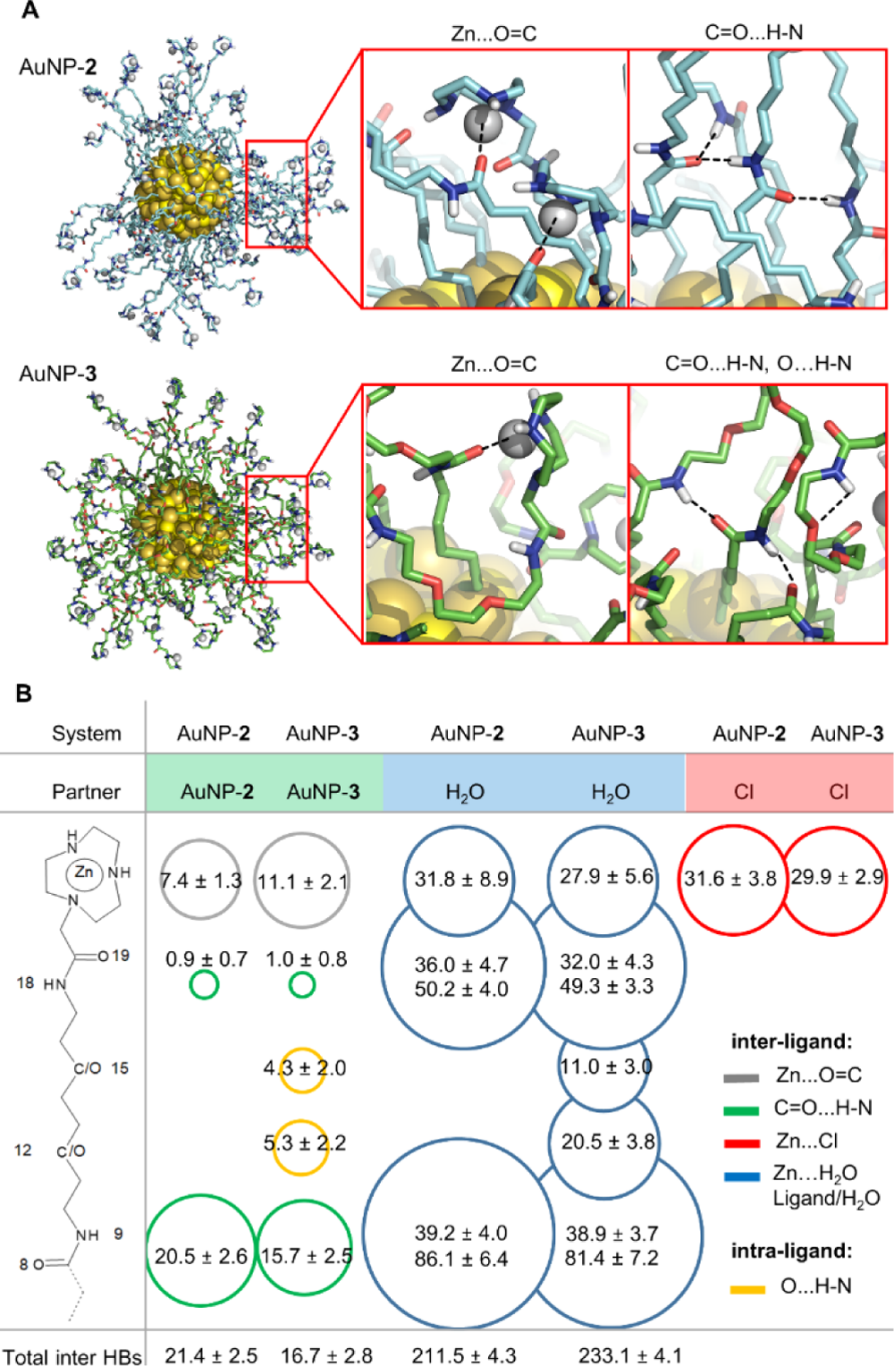
Inter- and intramolecular interactions of AuNP-**2** and AuNP-**3**. (A) Representative snapshot (at 100 ns of the simulation time) showing differently pre-organized monolayers of AuNP-**2** (in blue) and AuNP-**3** (in green), zooming into interligand Zn(II)-carbonyl oxygen (O8) interactions, multiple HBs between C=O8 and H–N9 HBs, and intraligand HBs between O12/O15 and H–N9/N18. (B) Average number of ligand–ligand interactions: with intraligand HBs between O12/O15 and amide groups (in orange), interligand HBs between C=O8 and N9–H (in green), and Zn–carbonyl oxygen (O8) interactions (Zn···O=C, in gray); average number of ligand–water HBs and contacts between Zn and water within 0.25 nm, in blue; and Zn···Cl interactions within 0.25 nm in red. There were no interligand interactions of O12/O15 with amide groups or between outer O19 and Zn ions.

The different extensions of the coating thiols influence the position of Zn ions chelated by the TACN crowns, overall located in between 1.5 and 3.5 nm (Figure 3A,C). Notably, the Zn curve has two maxima, where the peak at a distance shorter than 2.2 nm reflects bent thiols, with the TACN crown pointing toward the gold core (Figure 3C). As a consequence, Zn ions can interact with the oxygen of the inner amide group (O8) more easily in AuNP-**3** than in AuNP-**2** (11.1 ± 2.1 and 7.4 ± 1.3, respectively—see Figure 4, Table S2). Accordingly, cumulative RDFs (Figure 3A) show a higher number of Zn ions at closer distances around the gold core in AuNP-**3**.

The second peak for the Zn RDF is at a distance higher than 2.2 nm, thus indicating elongated conformations of the ligands. That is, the majority of Zn ions is located on the outer part of the monolayer of both nanoparticles (Figure 3C). The more extended thiols of AuNP-**2** resulted in a broad Zn distribution peak at a distance of 3.1 nm, while the less extended thiols in AuNP-**3** are found at 2.9 nm (Figure 3C). Finally, we note that Zn ions interact similarly with Cl ions in both systems (Figure 4B, 31.6 ± 3.8 interactions with Zn ions for AuNP-**2** and 29.9 ± 2.9 for AuNP-**3**).

**Figure 5:**
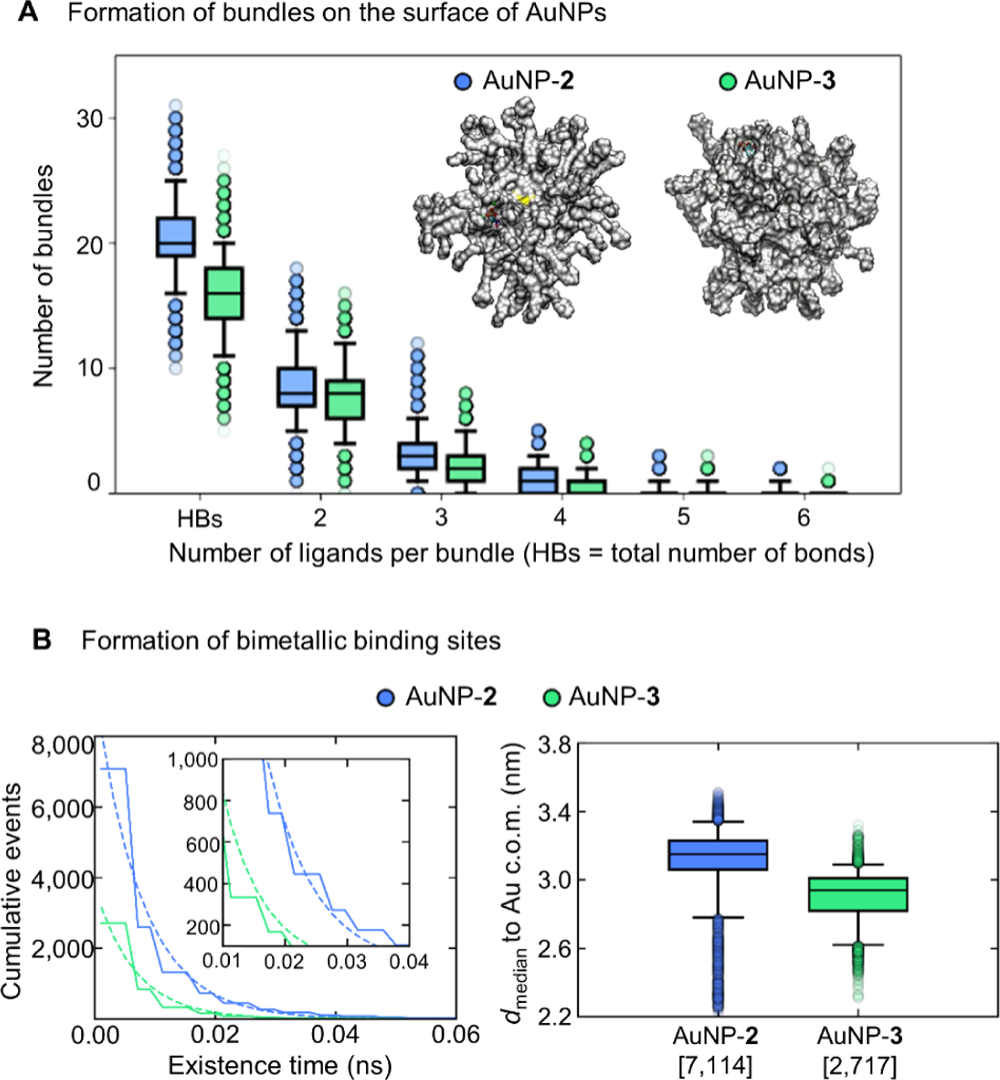
(A) Frequency of the formation of bundles—defined as more than two (up to six) coating ligands interconnected simultaneously by HBs between C8=O and H–N9 atoms of AuNP-**2** and AuNP-**3**. (B) Formation of bimetallic binding sites defined by a close proximity (0.50 nm) of two Zn ions. The graph (on the left) shows the cumulated number of events at different existence times. The data (solid lines) were fitted to a single exponential (dotted line) to compare the decay rate (λ) of both nanoparticles. The inset shows a close view of the cumulated events with an existence time between 0.01 and 0.04 ns. The boxplots (on the right) show the distributions of the median distance of bimetallic sites from the center of mass (c.o.m.) of the gold core. The square brackets on the *x*-axis show the total number of binding sites for each nanoparticle.

AuNP-**3** not only features more interaction between the Zn ions and the O8 amide oxygen but also several inter-ligand hydrogen bonds (HBs) between O12/O15 and the nearest amides, which are indeed possible only with these AuNPs (Figure 4). On the contrary, in AuNP-**2**, we observed more structured networks of HB chains that connect the inner amide group of three or four nearby coating ligands (Figure 5A). Such H-bond networks correlate with a close association of the thiols to form bundles^18^ (Figure 5A, inset).

Solvation water molecules penetrate at different levels of the monolayer (Table S2 and Figure S15). Indeed, they are found down to the region of highly packed inner alkyl chains reaching the C3–C6 region in the case of AuNP-**2**, while their penetration is limited to the C5–C6 region in the case of AuNP-**3** (Figure 3A). Water molecules form an extensive network of hydrogen bonds with the coating ligands. Most of these HBs are formed with the inner amide group (O8 and N9) of both nanoparticles, with 125.3 ± 9.7 HBs for AuNP-**2** and 120.3 ± 9.2 HBs for AuNP-**3** (Figure 4B), as well as with the outer amide group (N18 and O19), with 86.3 ± 6.7 and 81.3 ± 5.6 HBs in AuNP-**2** and AuNP-**3**, respectively. The major difference comes from the linker part, which can form HBs with water molecules only in AuNP-**3**. The hydrophilic PEG linkers of AuNP-**3** form 31.5 ± 4.8 HBs with water molecules (Figure 4B).

Taken together, these results confirm that in AuNP-**2** the coating ligands are more extended, and they tend to pack into H-bonded bundles, likely as a consequence of the greater hydrophobicity of the linker. Although both nanoparticles are similarly spherical (as indicated by the eccentricity values of 0.055 ± 0.005 for AuNP-**2** and 0.050 ± 0.003 for AuNP-**3**), bundling generates irregular canyons (i.e., “rifts”) within the coating monolayer of AuNP-**2** that facilitates the penetration of water molecules deeper toward the gold core. On the other hand, the oxygen atoms in the linker of AuNP-**3** form HBs both with the ligand amides and with water. These interactions favor thiol folding and ligand spacing (water bridges between ligands) and help to prevent bundling (Figure 5A).

### Bimetallic Binding Sites Are More Frequent and Long-Living in AuNP-**2** Compared to AuNP-**3**

At this point, we monitored the formation of the bimetallic binding sites able to mimic the well-recognized two-metal-ion enzymatic architecture for phosphate hydrolysis. Such bimetallic binding sites are here defined by two Zn ions in close proximity to one another. We also computed the distance of each bimetallic site from the gold core’s center, as well as the existence time of such bimetallic sites over the MD simulations (Figure S16). To compare both nanoparticles, we computed the population of binding sites (i.e., the cumulated number of sites) at different existence times in each AuNP (Figure 5B) and compared populations’ decay rates λ (ns^–1^) that reflect how fast the number of events decays with respect to their existence time (as specified in the Methods section).

In AuNP-**2**, where the monolayer organizes more often into bundles, the Zn bimetallic sites form more frequently. They also show a slightly longer existence time than in AuNP-**3** (Figure 5A,B). In detail, AuNP-**2** formed 128 “unique” bimetallic sites (defined by the proximity of two Zn ions within a threshold of 0.50 nm), where the term “unique” refers to a distinctive pair of Zn ions, out of the 60 Zn ions chelated by the thiols. Particularly, such bimetallic sites were formed 7,114 times, with an existence time ranging from 5 ps to 22.8 ns, where 472 binding sites lasted longer than 20 ps (λ = 131.0 ns^–1^, Figure 5B). The location of the bimetallic sites agrees with the Zn-rich region identified from our RDFs (with a median of 3.2 nm from the gold core’s center, Figure 5B). AuNP-**3**, on the other hand, formed only 80 unique bimetallic sites for 2717 times, with an existence time ranging from 5 ps to 9.9 ns. Here, only 93 binding sites lasted longer than 20 ps (λ = 150.4 ns^–1^, Figure 5B). The median distance of bimetallic sites in AuNP-**3** was 2.9 nm from the gold core’s center. Also, analysis using less tight thresholds of the Zn–Zn distance (i.e., 0.55 or 0.63 nm) showed a greater ability of AuNP-**2** to form bimetallic sites, compared to AuNP-**3** (Figure S17).

Thus, the diverse structural organization of the coating monolayer in AuNP-**2** and AuNP-**3** influences the position and the catalytic pre-organization of the Zn ions chelated to the TACN crowns. In AuNP-**2**, the Zn ions on the surface of the nanoparticle are more distant from the gold core, whereas in the more compact AuNP-**3**, not only the Zn-rich pseudo-phase is closer to the core but also there is a relevant population of Zn ions buried inside the monolayer. However, bimetallic sites are mostly located in both the nanoparticles at the level of the outer Zn-rich regions. The formation of transient bundles in AuNP-**2** thus seems to favor conformationally constrained structural regions where bimetallic binding sites can more easily form. Indeed, our simulations reveal that bimetallic sites form more frequently (2.6-fold) and with a longer existence time (5.1-fold long-lived sites) in AuNP-**2** than in the less catalytically efficient AuNP-**3**. Notably, the structure of these bimetallic sites matches with what was suggested in previous studies, which have shown that the ideal Zn–Zn distance for the coordination of both phosphoryl oxygen atoms of the substrate’s phosphate group ranges from 0.4 to 0.7 nm. As an example, Meyer et al.^19^ showed that a distance below 0.4 nm leads to the inactivation of the catalyst; whereas, Scrimin et al.^20^ concluded that a distance of 0.63 nm is the upper limit for the HPNP cleavage by AuNPs passivated with oligopeptide-based ligands.

**Figure 6:**
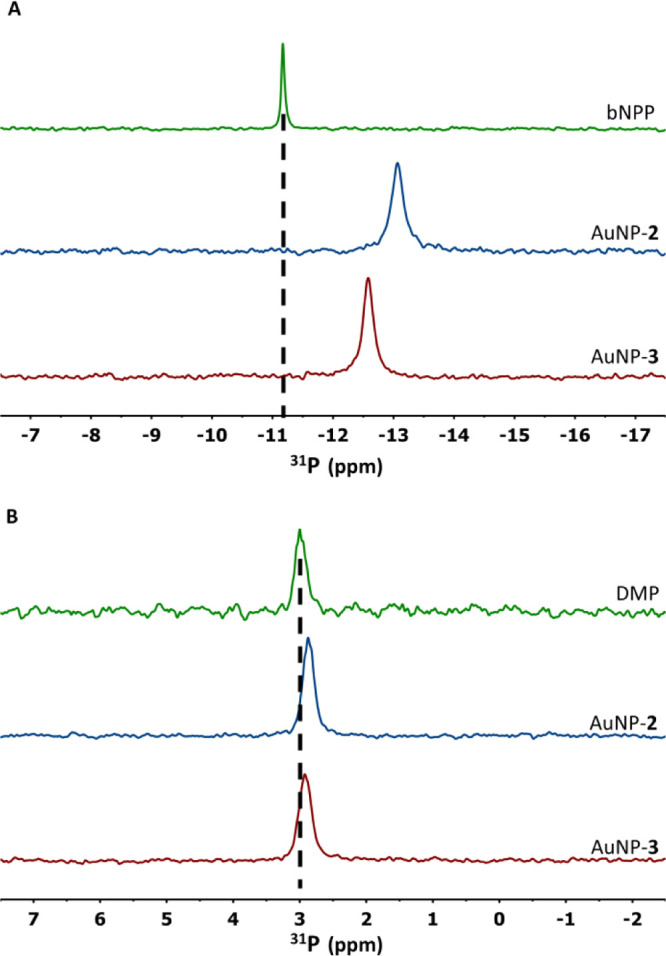
^31^P NMR spectra of (A) bNPP (bis-4-nitrophenylphosphate, 2.5 mM in D_2_O) alone (green spectrum) and in the presence of AuNP-**2** (blue) or AuNP-**3** (red); (B) DMP (dimethylphosphate, 2.5 mM in D_2_O), alone and in the presence of AuNP-**2** or AuNP-**3**. Conditions: [AuNP] = 71 μM, 25 °C.

In analogy with the approach used in the absence of the substrate, we characterized, in more detail, the interaction of phosphate ester substrates with Zn ions, in either AuNP-**2** or AuNP-**3**. To amplify the previous measurements with HPNP,^10a^ in these new experiments, we used and recorded the ^31^P NMR spectra of the poorly reactive diesters dimethylphosphate (DMP) and bis-4-nitrophenylphosphate (bNPP), with the nanoparticle (either AuNP-**2** or AuNP-**3**) and one equivalent of Zn with respect to the TACN units present (Figure 6). In both cases, the addition of the nanoparticles results in an upfield shift of the ^31^P signal as a consequence of the interaction of the phosphate oxygen atoms with the Zn ions.^21^ The effects are very small in the case of DMP and larger in the case of bNPP because the latter, being more hydrophobic, binds more strongly to the nanoparticles. With both substrates, however, the shift observed in the presence of AuNP-**2** is larger than that observed with AuNP-**3**. Because the affinity of the two nanoparticles for phosphate diesters is similar, the fraction of bound substrates should be the same. Hence, larger shifts can arise only from a different interaction of the phosphate groups with the Zn ions present in the monolayers.

Then, we further characterized the substrate binding to AuNP-**2** and AuNP-**3** with a new set of MD simulations. For both nanoparticles, each time we considered 10 HPNP molecules in solution (four 200 ns long MD replicas for the AuNP-**2**/substrate and the AuNP-**3**/substrate systems at 300.00 K as well as at 313.15 K, the temperature used in kinetic experiments; 3.2 μs of simulation time, in total).

Interestingly, these MD simulations revealed that the overall organization of the monolayer described earlier is not affected by the presence of the substrate. This is evident by looking at: (i) the compactness, or the shape of the nanoparticles (Table S3); (ii) the morphology of the monolayer, as indicated by the RDFs (see Figure S18); and (iii) the extent of intramolecular interactions: C=O8···Zn interactions stabilizing bent ligands and C=O8···H–N9 interactions interconnecting two or more ligands into the bundles (Table S3 and Figure S19).

Thus, we turned our attention to the analysis of substrate recognition and binding events. First, we found that HPNP coordinates to the Zn ions only through its scissile phosphate group, as there is no interaction with the NO_2_ of the *p*-nitrophenyl (PNP) leaving group. According to RDFs, phosphate groups of the HPNP molecules are closer to the gold core than the Zn atoms, confirming that the substrate is not bound to the surface of the monolayer but penetrates inside it, as a result of the concomitant charge pairing (and coordination) and hydrophobic interactions at play (Figure S20). Moreover, both AuNPs have a similar number of simultaneously bound substrates, via the coordination of the phosphate group to at least one Zn ion (4.9 ± 1.2 substrates in AuNP-**2** and 5.3 ± 1.4 substrates in AuNP-**3**).

**Figure 7:**
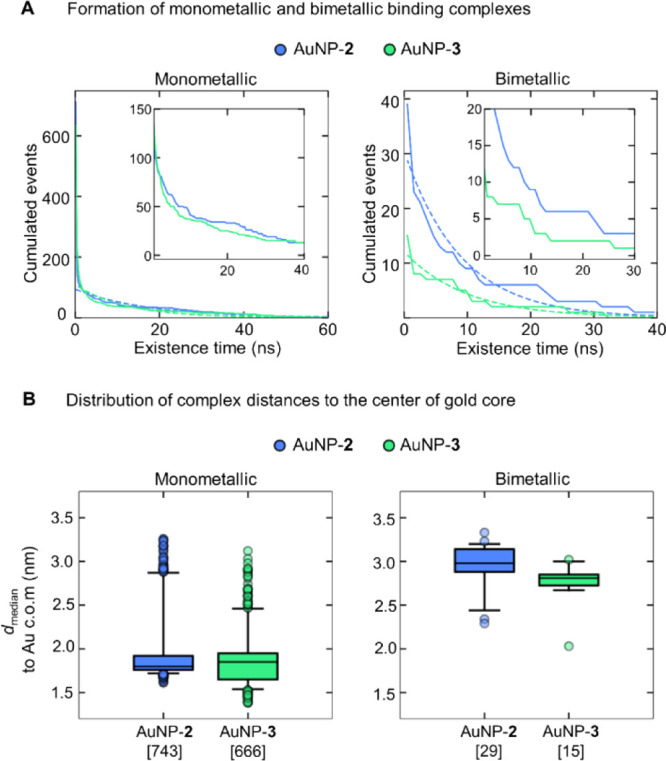
Formation of AuNPs/substrate monometallic and bimetallic binding complexes. (A) Graphs showing the decaying population of binding complexes (monometallic, on left; and bimetallic, on right) as the existence time increases. The data (solid lines) were fitted to a single exponential (dotted line) to compare the decay rate (λ) of both nanoparticles. The insets show a close view of the cumulated events with an existence time between 1 and 40 ns for monometallic complexes and between 1 and 30 ns for bimetallic ones. (B) Boxplots showing the distance distributions of binding complexes [median distance to the center of mass (c.o.m.) of the gold core, calculated from all the distances of the complex formation]. The square brackets on the *x*-axis show the total number of binding sites for each nanoparticle.

Then, we analyzed the ability of each nanoparticle to form mono- and bimetallic binding complexes with the substrate (Figures 7 and S21). The monometallic complex is formed when one or both oxygen atoms of the phosphate group (O4/O5) in HPNP coordinate(s) to a single Zn ion (within 0.25 nm) and no additional Zn is involved in HPNP binding. With these parameters, we found that both nanoparticles form monometallic complexes. In AuNP-**2**, we observed a total of 743 monometallic complexes with an existence time spanning from 5 ps to 394.7 ns and a decay rate of λ = 0.06 ns^–1^ (Figure 7A). Instead, AuNP-**3** formed 666 monometallic complexes with an existence time spanning from 5 ps to 373.6 ns and a decay rate of λ = 0.08 ns^–1^ (Figure 7A). Notably, AuNP-**2** forms more monometallic complexes than AuNP-**3**, and they also tend to be longer lived, as indicated by the slightly lower decay rate (0.06 ns^–1^ in AuNP-**2** and 0.08 ns^–1^ in AuNP-**3**).

At this point, we examined in more detail the formation of bimetallic AuNP/HPNP complexes, defined by the simultaneous coordination of two oxygen atoms of the phosphate group (O3/O4) in HPNP that are within a distance of 0.25 nm from the two different Zn ions in the monolayer (Figure 7A). Here, we noticed that the Zn–Zn internuclear distance is similar in all the bimetallic complexes, that is, 0.53 ± 0.02 nm in AuNP-**2** and 0.54 ± 0.02 nm in AuNP-**3** (Figure S22). Importantly, we found that AuNP-**2** forms more bimetallic binding complexes than AuNP-**3**, although the duration of the events follows a similar distribution decay in both nanoparticles. In total, AuNP-**2** formed 39 bimetallic complexes, with an existence time spanning from 5 ps to 39.4 ns, where 28 complexes lasted longer than 100 ps (λ = 0.11 ns^–1^, Figure 7A). On the other hand, AuNP-**3** formed 15 bimetallic complexes in total, with an existence time spanning from 5 ps to 31.0 ns. Here, only 11 complexes lasted longer than 100 ps (λ = 0.10 ns^–1^, Figure 7A).

Overall, there are only small differences in the location of the monometallic complexes in both nanoparticles (with median distances at 1.8 and 1.9 nm to the gold core’s center of AuNP-**2** and AuNP-**3**, respectively; Figure 7B). Bimetallic complexes are, on the other hand, slightly farther away from the gold core in AuNP-**2** (with a median distance of 3.0 nm) than in AuNP-**3** (a median of 2.8 nm; Figure 7B). Monometallic complexes are hence located deeper in the monolayer than the bimetallic ones. Bimetallic sites are in the outer region likely due to the difficulty of inducing the bending of a double-anchored structure.

As in the simulations performed in the absence of HPNP, water penetrates more deeply in the monolayer of AuNP-**2** than that of AuNP-**3** because of the presence of solvent-exposed rifts in the former (Figure S20). In detail, 989 water molecules are found within 2.5 nm from the particle center in AuNP-**2** and 948 in AuNP-**3**. However, a different trend is observed when the solvation of the outer portion of the monolayer, where most of the bimetallic complexes are located, is examined. The region included between 2.5 and 3.2 nm accommodates 1978 water molecules in the case of AuNP-**2** and 2009 in the case of AuNP-**3**. Because rifts are still present and are even more relevant in this region, the slightly larger number of water molecules found in AuNP-**3** is likely due to the solvation of the oligoethylene oxide spacers discussed earlier.

**Figure 8:**
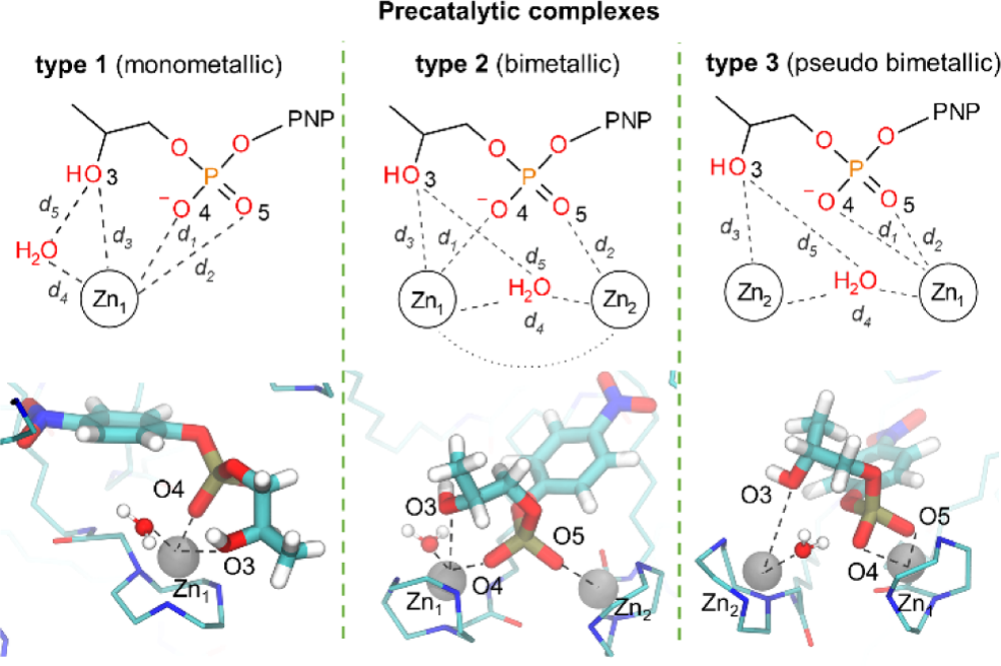
Formation of precatalytic complexes with HPNP, defined as a metric where at least three distances between the involved oxygen atoms and Zn ion(s) (*d*_1_, *d*_2_, and *d*_3_) are below a defined threshold of 0.25 nm. The distances *d*_4_ and *d*_5_ indicate the active state of such precatalytic complexes, that is, when the water molecule binds to the Zn ion (*d*_4_ < 0.20 nm) and when the nucleophile is ready for the activation (*d*_5_ < 0.25 nm).

**Figure 9:**
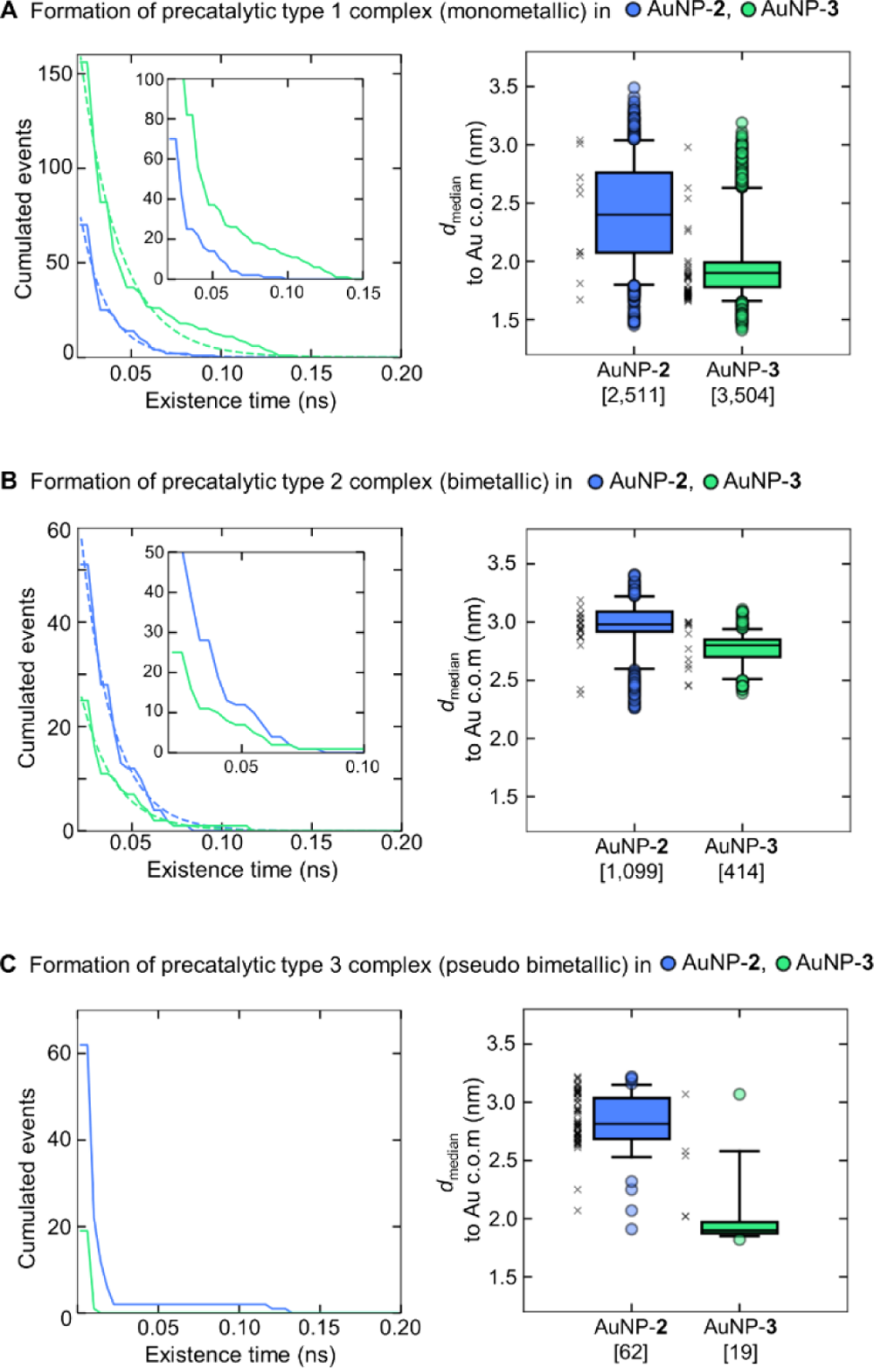
Formation of three different precatalytic complexes inAuNP-**2** (in blue) and AuNP-**3** (in green); monometallic precatalytic type 1 complex (A), bimetallic precatalytic type 2 complex (B), and pseudo-bimetallic precatalytic type 3 complex (C). The graphs on the left show the decaying population of precatalytic complexes as the existence time increases. The data (solid lines) were fitted to a single exponential (dotted line) to compare the decay rate (λ) of both nanoparticles. The insets in A and B show a close view of the existence time (between 0.02 and 0.15 ns in A and 0.02 and 0.10 ns in B). The fitting of the type 3 complex is omitted due to the minimal number of points available. The boxplots on the right show the distance distributions of precatalytic complexes (as a median distance to the center of mass (c.o.m.) of the gold core calculated from all the distances during the complex formation). The square brackets on the *x*-axis show the total number of binding sites for each nanoparticle. Black crosses represent the solvated precatalytic complexes in the presence of at least one Zn-coordinated water molecule.

Refining the analysis of the Zn complexes bound to HPNP, we found three different precatalytic complexes, that is, configurations that can result in the cleavage reaction (as specified in the Methods section and caption of Figure 8). The most frequent one was the monometallic type 1 complex, which is formed when all the reactants are centered on one single Zn ion (Figure 9A and S23). Such a monometallic arrangement of the type 1 precatalytic complex in AuNP-**2** is formed 2,511 times during 700 ns, with an existence time of up to 95 ps. Here, only 70 complexes lasted longer than 20 ps (λ = 68.4 ns^–1^, Figure 9A). In AuNP-**3**, the monometallic precatalytic complexes are formed more frequently, that is, 3,504 times, with an existence time of up to 140 ps. Here, 156 complexes lasted more than 20 ps. Importantly, in AuNP-**3**, the monometallic precatalytic complexes are formed 1.4-fold more often than in AuNP-**2**, and these complexes also tend to be longer lived, with the decay rate decreasing from 68.4 ns^–1^ in AuNP-**2** to 46.3 ns^–1^ in AuNP-**3** (Figure 9A). The location of monometallic precatalytic complexes significantly differs in each nanoparticle (Figure 9A); whereas, AuNP-**2** forms such complexes at a median distance of 2.4 nm from the gold core’s center (with solvated complexes distributed in multiple regions of the monolayer), AuNP-**3** forms monometallic complexes at a median distance of 1.9 nm and with the majority of solvated complexes located in the inner part of the monolayer (Figure 9A).

Then, we identified the bimetallic type 2 complex, where both oxygen atoms of the substrate’s phosphate group (O4/O5) were simultaneously bound to two different Zn ions (*d*_1_ and *d*_2_ < 0.25 nm), while one of the Zn ions also coordinated the oxygen of HPNP’s hydroxyl group (O3 with *d*_3_ within 0.25 nm, Figure 8). Here, AuNP-**2** forms 2.7-fold more bimetallic complexes than AuNP-**3** (Figure 8). In detail, AuNP-**2** forms 1,099 complexes, with an existence time of up to 80 ps. Here, 51 complexes lasted more than 20 ps. AuNP-**3**, on the other hand, formed only 414 complexes in total, with an existence time of up to 115 ps and with 25 complexes lasting more than 20 ps. Interestingly, the duration of the type 2 precatalytic complexes was comparable for both nanoparticles (λ = 57.5 ns^–1^ in for AuNP-**2** and λ = 53.9 ns^–1^ for AuNP-**3**, Figure 9B). Similarly for both AuNPs, one water molecule would occasionally bind to one of the Zn ions (19 times in AuNP-**2** and 12 times in AuNP-**3**). The location of bimetallic type 2 complexes is in both nanoparticles in the outer region of the monolayer, that is, at median distances of 3.0 and 2.8 nm for AuNP-**2** and AuNP-**3**, respectively. The Zn–Zn internuclear distance is similar in all the bimetallic precatalytic complexes, that is, 0.55 ± 0.02 nm in AuNP-**2** and 0.54 ± 0.03 nm in AuNP-**3** (Figure S24).

Finally, we also identified the formation of a “pseudo-bimetallic” type 3 precatalytic complex. Here, only one of the Zn ions directly coordinates one of the oxygen atoms (O4/O5) of the HPNP phosphate group (*d*_1_ and/or *d*_2_ < 0.25 nm), while the second Zn ion interacts only with O3 of the HPNP’s hydroxyl group (*d*_3_ < 0.25 nm, as indicated in Figure 8). This complex is more frequently formed in AuNP-**2** than in AuNP-**3**. AuNP-**2** forms 62 type 3 precatalytic complexes in total, with an existence time of up to 130 ps, where five complexes lasted longer than 20 ps. AuNP-**3** forms only 19 type 3 complexes, with an existence time spanning from 5 to 10 ps (Figure 9C). We note that most of the precatalytic type 3 complexes (i.e., 59) in AuNP-**2** contain at least one Zn-coordinated water molecule, and only 5 complexes were solvated in AuNP-**3** (as indicated by black crosses in Figure 9C). This is in line with the respective location of such precatalytic complexes. In AuNP-**2**, these form mostly on the surface of the monolayer (at a median distance of 2.8 nm to the gold core’s center), whereas the position of such complexes in AuNP-**3** is in the inner part of the monolayer (at a distance of 1.9 nm; Figure 9C).

Precatalytic states do not often transit between each other (Figure S25). Interestingly, AuNPs also differ in a possible sequence of the steps to be catalytically activated along the reaction coordinate. In particular, we found two different pathways in AuNP-**2**: one that connects monometallic binding complexes to the bimetallic type 2 complex and a second less probable path that connects the bimetallic binding complexes to the monometallic type 1 complex. In AuNP-**3**, all states are in general less interconnected, there is no direct transition between precatalytic states. The bimetallic precatalytic type 2 complex is, here, totally disconnected from the others as it is formed only from bimetallic binding complexes (see Figure S25).

### Catalytic Strategy of AuNP-**2** versus AuNP-**3**

Importantly, simulations performed with HPNP show that the mean number of substrate molecules bound to the monolayer at every timestep of the simulation is similar for the two nanozymes (4.9 ± 1.2 in AuNP-**2** and 5.3 ± 1.4 in AuNP-**3**). These data suggest that both nanoparticles have a comparable ability to bind to the substrate, which is in line with their similar *K*_M_ values experimentally determined (0.40 nM for AuNP-**2** and 0.38 nM for AuNP-**3**).^10a^

The analysis of the bimetallic sites in the two nanoparticles revealed relevant differences in the formation and features of such chemical entities. Bimetallic sites are more frequently found in AuNP-**2**, compared to AuNP-**3**. In particular, the amount of long lasting bimetallic sites is 5.1-fold larger in AuNP-**2** than in AuNP-**3**. The clustering of the thiols in bundles results in a better pre-organization for catalysis. That is, the larger number of bimetallic sites found in AuNP-**2** results, as one may expect, in a larger number of bimetallic complexes with the substrate and bimetallic precatalytic sites. The different interactions, resulting from the MD simulations, between the substrate and the metal ions in AuNP-**2**, compared to AuNP-**3**, are also confirmed by ^31^P NMR experiments. Indeed, the substrate affinity of both nanoparticles being similar, the larger shifts of the phosphorus signals obtained with AuNP-**2** can be explained by a different coordination environment.

**Figure 10:**
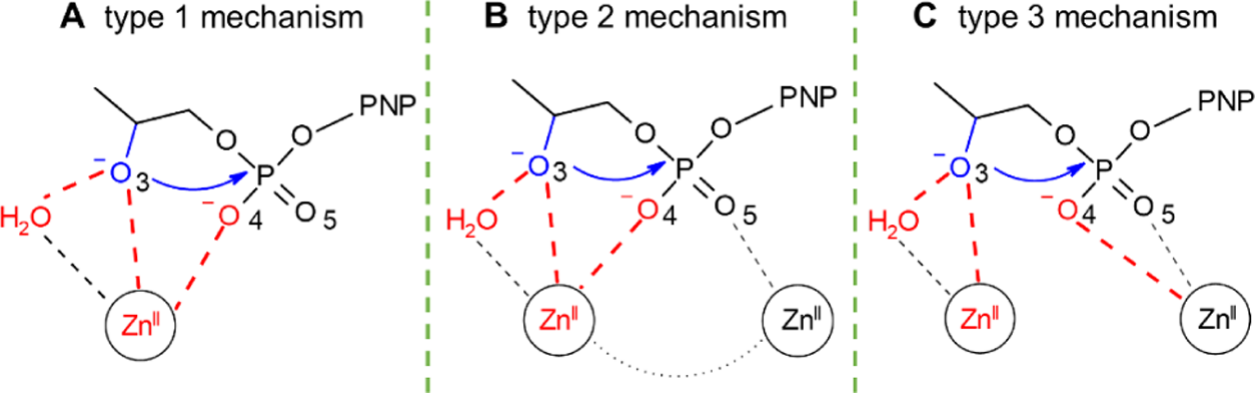
Possible catalytic mechanisms for the cleavage of the phosphate bond in HPNP by AuNP-**2** and AuNP-**3**. (A) Mechanism with one single metal ion and (B,C) mechanism with two metal ions. Functional groups directly involved in catalysis are in red and the nucleophilic group is in blue.

The precatalytic configurations found in these nanozymes can in principle lead to substrate transesterification through the intermolecular nucleophilic attack of the hydroxyl group on the phosphorus atom, followed by the release of 4-nitrophenol (Figure 10). The presence of a large number of precatalytic sites in AuNP-**2** reflects well the reactivity difference observed in the experiments with these two AuNPs. In addition, our MD simulations confirm the early experimental observation of a decreased solvation of the Zn(II)-rich region of AuNP-**2**, which could also contribute to the greater catalytic effectiveness of these AuNPs.^10a^ We note that the quantification of the exact catalytic effect of such mechanistic and structural aspects in AuNP-**2** versus AuNP-**3** will, however, require quantum mechanical computations for catalysis.

In summary, this detailed comparison between AuNP-**2** and AuNP-**3** thus shows that one single and minimal structural modification of the coating thiol can affect significantly and in a nonintuitive way the structure and the properties of the monolayer. In particular, we found significant differences in the formation of the optimal precatalytic centers in these nanonucleases, with enhanced bimetallic cooperativity in AuNP-**2**—mostly as in the type 2 complex, which is reminiscent of the two-metal-ion mechanism for nucleic acid-processing enzymes (Figure 10B).^7^

## Conclusions

We used μs-long classical MD simulations coupled with NMR experiments for a detailed comparison between AuNP-**2** and AuNP-**3**, which are two structurally similar nanozymes that, however, have a different efficiency for phosphodiester cleavage. We explain how the insertion of a hydrophobic alkyl chain in AuNP-**2** (instead of a hydrophilic PEG chain in AuNP-**3**) causes a different extent of solvation and stabilization within the monolayer. We propose that a specific balance of intraligand, interligand, and solvent–ligand interactions produces a more extended conformation and the bundling of the coating ligands in AuNP-**2**. Spatial proximity and correlated dynamics of the ligands resulting from this bundling promote the formation of a larger number of long lasting bimetallic sites that can efficiently process the substrate HPNP. Thus, our simulations suggest that the more catalytically efficient AuNP-**2** is indeed able to form precatalytic bimetallic and pseudo-bimetallic complexes more often than AuNP-**3**, which instead preferentially forms monometallic sites—in agreement with our experimental evidence. MD simulations also support the early hypothesis that the catalytic effectiveness of these bimetallic sites could be increased by the lower local polarity at the reaction site because we found a smaller solvation of the Zn-rich monolayer region of AuNP-**2** with respect to AuNP-**3**. These mechanistic findings contribute to explain why the monolayer of AuNP-**2** is more efficient in processing the substrate HPNP.

Taken together, our results show that the exact structure and dynamics of the coating thiols of such nanoparticles is crucial to finely regulate their catalytic efficiency. Our data also suggest that efficient nanonucleases preferentially form a (bi)metal-aided precatalytic site for phosphate ester hydrolysis (type 2 complex, Figure 10B). Notably, this catalytic strategy is reminiscent of the two-metal-ion mechanism for nucleic-acid processing used by many natural metalloenzymes, including polymerases, nucleases, and topoisomerases.^7^ These mechanistic insights reinforce the parallelism of such nanozymes with proteins, advocating for the rational design of nanonucleases with enhanced efficiency.

## Methods

### Nanoparticle Synthesis

Functionalized AuNPs were prepared following a two-step procedure previously reported.^22^ First, a solution of HAuCl_4_·3H_2_O was extracted with a solution of tetraoctylammonium bromide (2.5 equiv) in N_2_-purged toluene. Then, dioctylamine (20 equiv to obtain ∼2 nm nanoparticles) was quickly added under vigorous stirring. When the color of the solution faded (2 h), the mixture was cooled to 0 °C and an aqueous solution of NaBH_4_ (10 equiv) was added. After 2 h of ageing, the water was removed, and a solution of the desired thiol (2 equiv) in methanol was added. After overnight stirring at room temperature, the so-obtained nanoparticles were collected by centrifugation, dissolved in a small amount of methanol, and precipitated again in ethyl acetate and diethyl ether. This procedure was repeated four times. The resulting AuNPs were further purified by a gel permeation chromatography with Sephadex LH-20. AuNP-**2** and AuNP-**3** were characterized by transmission electron microscopy (TEM), thermogravimetric analysis (TGA), and ultraviolet–visible spectroscopy (UV–vis) analyses. The formation and composition of the monolayer were confirmed by NMR analysis (see the Supporting Information for the complete characterization).

TEM images (Figures S2 and S5) were recorded on a JEOL 300 PX electron microscope. One drop of the AuNP solution (1 mg/mL) was placed on the sample grid and the solvent was removed by absorption on a filter paper.

TGA was performed on a Q5000 IR model TA instrument (∼0.5 mg AuNP, ramp at 10 °C/min from 100 to 1000 °C) under a continuous air flow (Figures S4 and S7).

UV–vis spectra and kinetic traces were recorded on a Cary 50 spectrophotometer equipped with thermostated multiple cell holders (Figure S8).

### NMR Experiments

The ^13^C relaxation times (*T*_1_) were measured with a standard inversion recovery pulse sequence on a Bruker AVIII HD 400 instrument operating at 400.13 MHz (^1^H) and 100.61 MHz (^13^C) equipped with a 5 mm DUL probe fort the direct observation of the ^13^C nucleus (Figures S9–S12). ^1^H and ^31^P NMR and bi-dimensional experiments were performed on a Bruker AVIII 500 instrument operating at 500.13 MHz (^1^H) and 202.46 MHz (^31^P), equipped with a 5 mm z-gradient BBI probe (Figure 6).

### Computational Models

The three-dimensional model of each nanoparticle is based on the Au_144_(SR)_60_ structure,^13^ which comprises 60 mercaptobenzoic acids grafted to the Au_144_ core via sulfur atoms. The *p*-mercaptobenzoic acids were substituted by our coating ligands. The coating thiols consist of three building blocks: (1) an octanamide group attaching the ligand to the gold core via a sulfur atom, (2) the long flexible linker, acetylaminooctyl in AuNP-**2** and acetylamino-diethoxyethyl in AuNP-**3**, and (3) TACN crown chelating Zn ions. The individual building blocks were parametrized by the general amber force field (GAFF).^23^ In particular, we adopted a force-field topology database building approach as developed in RedServer.^24^ This is a combinatory approach; first, for each building block, two conformers were generated using the Maestro suite.^25^ Second, atomic charges were derived from the RESP fitting procedure^26^ at the HF/6-31G* level in Gaussian-09^27^ (for more details see the RedServer webpage).^24^ Finally, the coating ligand was built by combining the parametrized building blocks. Zn ions were modeled at a distance of 2.1 Å from each N atom of the chelating TACN crown using 12–6 Lennard-Jones parameters.^28^ A charge transfer to a Zn of 0.19e from each N of the crown was included. These estimations were based on the DFT: TPSS/TZVP-optimized model consisting of the CH3-TACN unit, the Zn ion, and one water molecule coordinated to the Zn. The final AuNPs were assembled via the NanoModeler webserver.^14^ NanoModeler combined the parameters for the coating ligands and Zn ions (described above) with the van der Waals parameters for gold atoms^29^ and AMBER compatible parameters for distances, angles, and dihedrals to keep the Au_144_/monolayer interface (staples) stable.^30^ The HPNP substrate was first optimized at the DFT-D3:BLYP/DZVP level and then parametrized by GAFF^23^ and RESP charges^26^ at the HF/6-31G* level.

### MD Simulations

At first, AuNP-**2** and AuNP-**3** models (∼4800 atoms) were minimized in vacuum via the steepest descent algorithm. The minimized systems were put into a rhombic dodecahedron simulation box, ensuring a minimum distance between the solute and the box edges of at least 1.6 nm. The boxes were, then, filled up with TIP3P^31^ explicit water molecules (around 27,000 molecules). For the AuNP-**2**/substrate and AuNP-**3**/substrate systems, 10 molecules of the HPNP substrate were added around each nanoparticle ensuring no initial contact with the coating monolayer. All systems were neutralized by substituting water molecules with Cl^–^ ions and the second minimization was applied to relax the solvent molecules and ions around the solute. The systems were, then, thermalized from 0 to 300 K with a canonical *NVT* simulation of 0.5 ns coupling the nanoparticle and the solvent with the ions to a V-rescale thermostat^32^ (τ_T_ = 0.1 ps^–1^). Then, we switched to the *NPT* statistical ensemble, where the systems were pressurized (*P* = 1 bar) for 0.5 ns in the *NPT* statistical ensemble (*P* = 1 bar), coupling the systems with a Parrinello–Rahman barostat^33^ (τ_P_ = 2 ps^–1^). Consequently, each system was fully equilibrated for 200 ns in the *NPT* (*P* = 1 bar, *T* = 300 K) statistical ensemble. After this initial phase, the systems were ready for productive runs in the *NPT* (*P* = 1 bar, *T* = 300 K) statistical ensemble. All bonds were constrained with LINCS,^34^ allowing a time-step of 2 fs. Periodic boundary conditions were applied to the systems in all directions. A smooth particle mesh Ewald (PME) method^35^ was used to evaluate long-range electrostatic interactions with a Fourier grid spacing of 1.6 Å, and a cutoff of 12 Å was used to account for the short-ranged nonbonded interactions.

Overall, we collected ∼5.2 μs of MD trajectories, specifically (i) 200 ns long MD simulations for AuNP-**2** and AuNP-**3** in the absence of Zn ions, where we collected coordinates of the systems every 2 ps to calculate ^13^C *T*_1_ relaxation times; (ii) a set of four 200 ns long replicas at 300 K for each of four different systems (AuNP-**2**, AuNP-**3**, AuNP-**2**/substrate, and AuNP-**3**/substrate) with the presence of Zn ions; and (iii) a set of four 200 ns long replicas of the AuNP-**2**/substrate and AuNP-**3**/substrate systems (with the presence of Zn ions) at the temperature used in kinetic experiments (313.15 K).^10a^ In all simulations in the presence of Zn ions, coordinates of the systems were collected and analysis performed every 5 ps. All the reported analyses refer to the production runs after ∼25 ns of the simulations, where the root mean square deviation (rmsd) of the AuNP’s atoms reached a plateau, ensuring the structural stability of the systems (Figures S25 and S26). All MD simulations were performed using the GROMACS-v5.1.4 package.^36^

### Calculation of *T*_1_ Relaxation times of ^13^C Nuclei

The spin-lattice relaxation time (*T*_1_) were calculated using the Lipari–Szabo approach^18^ and as described in ref (12a), which considers the internal and overall motions independently. For the internal motions, at first, we fitted the AuNP structure to the fully equilibrated structure at time *t* = 200 ns of the first NPT simulation. The autocorrelation functions for each CH vector were extracted from the trajectories, considering a time step of 2 ps. The autocorrelation functions were, then, fitted by a two-exponential fit. For the overall motions, we calculated the rotational diffusion coefficient from the Stokes–Einstein equation (*D*_rot_ = *k*_B_*T*/8πη*r*^3^), where *T* is the absolute temperature, *k*_B_ is the Boltzmann constant, η is the dynamic viscosity of the solvent, and *r* is the effective hydrodynamic radius of the nanoparticles, which we considered to be 1.1 times the average radius of gyration.

### Classification of Precatalytic Complexes

The precatalytic complexes are a subset of the monometallic and bimetallic binding complexes. Such precatalytic complexes are defined by the following metric: the three distances (*d*_1_, *d*_2_, and *d*_3_) between the oxygen atoms (O3, O4, and O5) and one/two Zn ion(s) (i.e., Zn_1_ and Zn_2_, as indicated in Figure 7) are below 0.25 nm. Solvated precatalytic complexes meet the condition of a Zn-bound water molecule within 0.20 nm (*d*_4_ distance, i.e., Zn_1_–O). The *d*_5_ distance (O–O3) indicates the possible activation of the nucleophile by one Zn-bound water, located at the distance <0.25 nm. The precatalytic type 1 complex (monometallic) is formed when all the reactants are placed around a single Zn ion. The distances *d*_1_, *d*_2_, and *d*_3_ between Zn1 and both phosphate’s oxygen atoms (O4 and O5), together with hydroxyl’s oxygen (O3) are below 0.25 nm. No other Zn ion is involved in HPNP binding. The precatalytic type 2 complex (bimetallic complex) is formed when both oxygen atoms of the substrate’s phosphate group (O4 and O5) are bound to two different Zn ions, simultaneously (*d*_1_ and *d*_2_ < 0.25 nm), while one of the Zn ions coordinates the oxygen of the HPNP’s hydroxyl (O3 with *d*_3_ within 0.25 nm). The precatalytic type 3 complex (pseudo-bimetallic) is formed when one of the Zn ions directly coordinates the oxygen atoms O4 and/or O5 of the phosphate group (with *d*_1_ and/or *d*_2_ within 0.25 nm), while the second Zn interacts only with the O3 of the hydroxyl group (*d*_3_ within 0.25 nm).

### Trajectory and Statistical Analyses

GROMACS-v5.1.4 package modules^36^ have been used for a trajectory processing and common data analysis. For the detection of binding sites and binding/precatalytic complexes, we used the Python programing language with the MDAnalysis^37^ library. All scripts are available at github.com/cebasfu93/MD_Analysis/blob/master/Nanozymes.

For the analysis of the existence time of the binding sites or binding/precatalytic complexes), we computed their population (i.e., the cumulated number of sites/complexes) at different existence times. We found that such population decays according to a single exponential equation: *N*(*t*) = *N*_0_ e^–λ*t*^, where *N*_0_ is the total number of events, *N*(*t*) is the number of events with an existence time longer than *t* (ns), and λ is the decay rate constant (ns^–1^). That is, the decay rate constant reflects how fast the number of events decays with respect to their existence time.

As for the analysis of the location of the binding sites and precatalytic complexes within the monolayer, we tracked the distance between the Zn ions and the gold atoms’ center of mass and then we extracted the median value for each of the sites identified. The distances’ distributions were compared using boxplots in which the boxes extend from the 25^th^ to the 75^th^ quantile, and the whiskers reach the 5^th^ and 95^th^ quantile. The boxes extend from the 25^th^ to the 75^th^ quantile, and the whiskers go from the 5^th^ and 95^th^ quantile, with a median (50^th^ quantile) as a black line.

## Supporting Information Available

Nanoparticles’ experimental characterization (TEM micrographs, size distribution, TGA curves, UV–vis absorbance spectra, ^1^H and ^13^C NMR spectra, ^1^H–^1^H COSY, and ^1^H–^13^C HSQC spectra) and MD-derived characteristics (C–H bond RCFs, ^13^C *T*_1_ relaxivities, contacts and HBs, solvation, RDFs from all MDs, quantitative comparison of simulations, formation of binding sites/complexes and precatalytic complexes, formation of bimetallic sites using less tight criteria, formation of bundles in AuNPs/sub, transition matrix, and rmsds) (PDF)
