# Supplementary material for: On the Metal-Aided Catalytic Mechanism for Phosphodiester Bond Cleavage Performed by Nanozymes
Source: ACS Catal. 2021 Jul 2;11(14):8736–48. doi: 10.1021/acscatal.1c01215 (PMC8397296; doi:10.1021/acscatal.1c01215)
Supplement: Supplementary file 1 — cs1c01215_si_001.pdf [file cs1c01215_si_001.pdf]

SUPPORTING INFORMATION

On the Metal-Aided Catalytic Mechanism for Phosphodiester Bond Cleavage Performed by Nanozymes

Adam Pecina,[†] Daniele Rosa-Gastaldo,[‡] Laura Riccardi,[†] Sebastian Franco-Ulloa,[†] Emil Milan,[‡] Paolo Scrimin,[‡,*] Fabrizio Mancin[‡,*] and Marco De Vivo[†,*]

[†] Laboratory of Molecular Modeling and Drug Discovery, Istituto Italiano di Tecnologia, Via Morego 30, 16163 Genoa, Italy
[‡] Dipartimento di Scienze Chimiche, Università di Padova, Via Marzolo 1, 35131 Padova, Italy

*** Corresponding Authors**
Paolo Scrimin: paolo.scrimin@unipd.it
Fabrizio Mancin: fabrizio.mancin@unipd.it
Marco De Vivo: marco.devivo@iit.it

1. Nanoparticles' experimental characterization

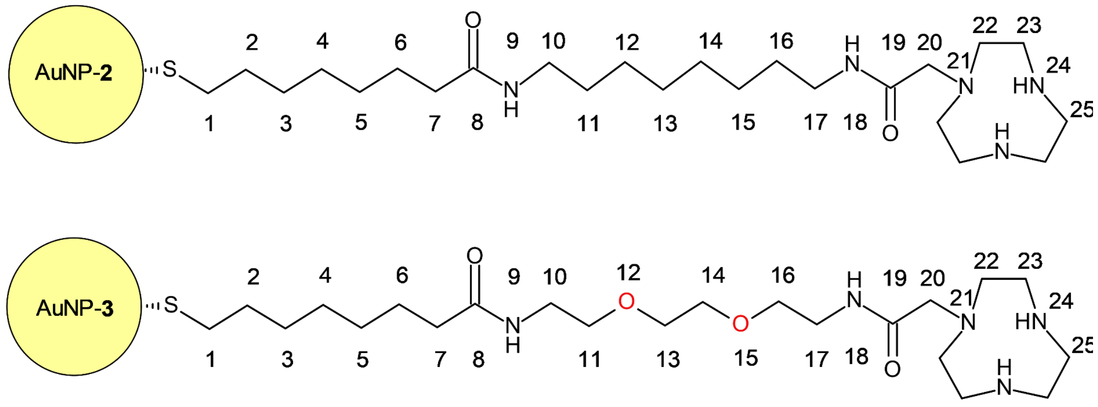

Figure S1. Coating ligands of AuNP-**2** and AuNP-**3**.

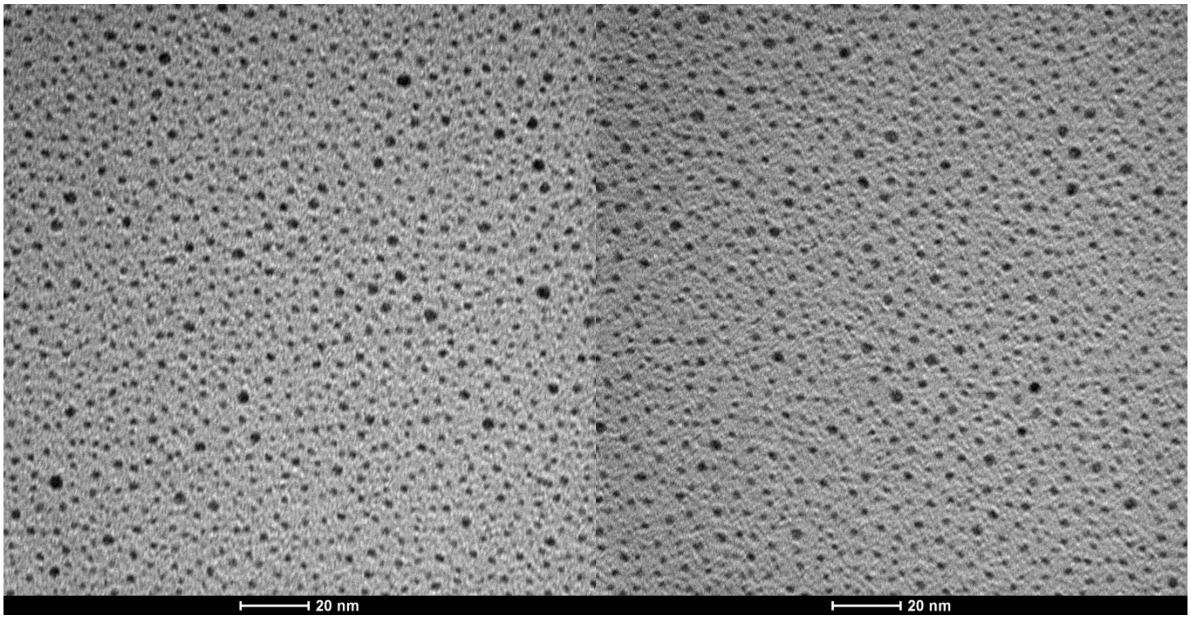

Figure S2. TEM micrographs of AuNP-**2**.

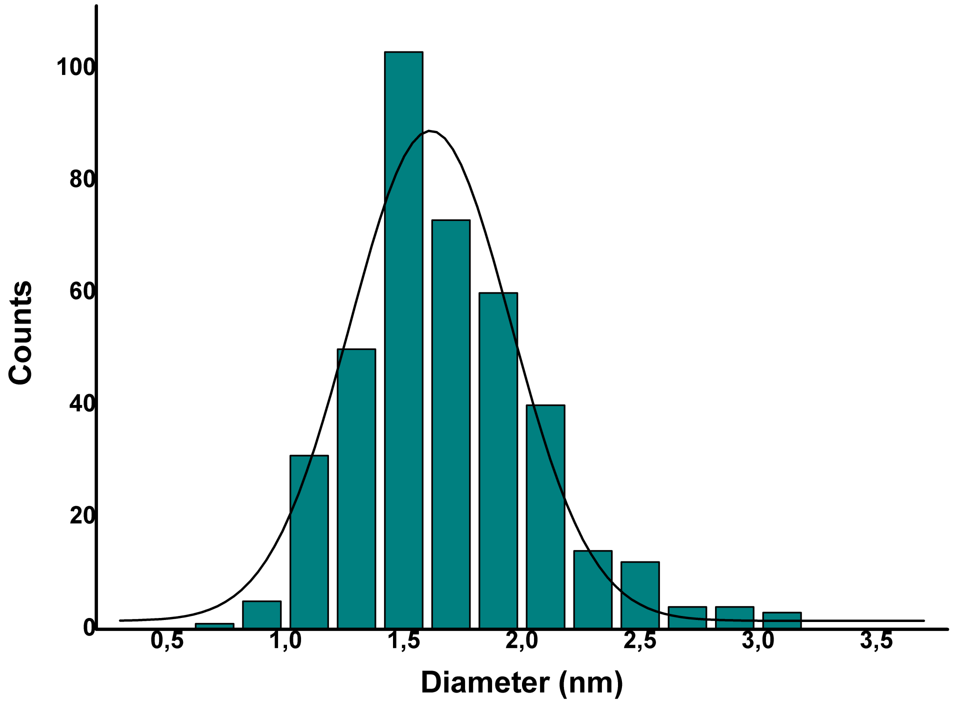

Figure S3. Size distribution of AuNP-**2** (d = 1.6 nm, σ = 0.3 nm).

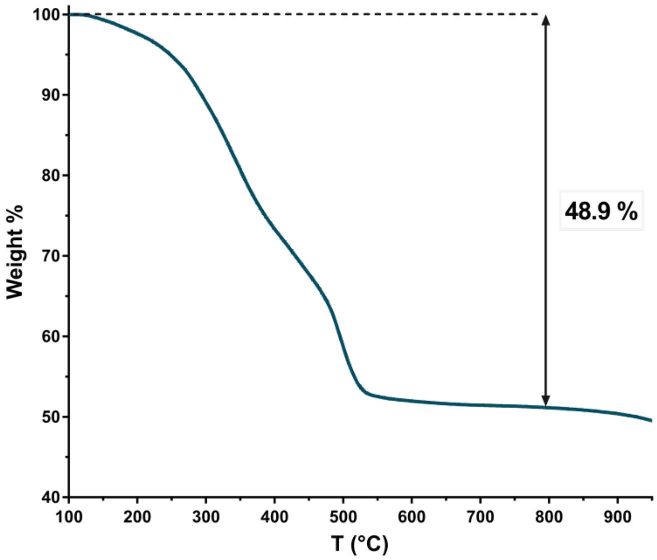

Figure S4. TGA analysis of AuNP-**2** (ramp 10 °C/min from 100 to 1000 °C, 0.5 mg of AuNPs, air atmosphere). From the combination of TEM and TGA data the average formula for AuNP-**2** is $Au_{127}SR_{51}$ with a thiol footprint of 0.16 nm^2.

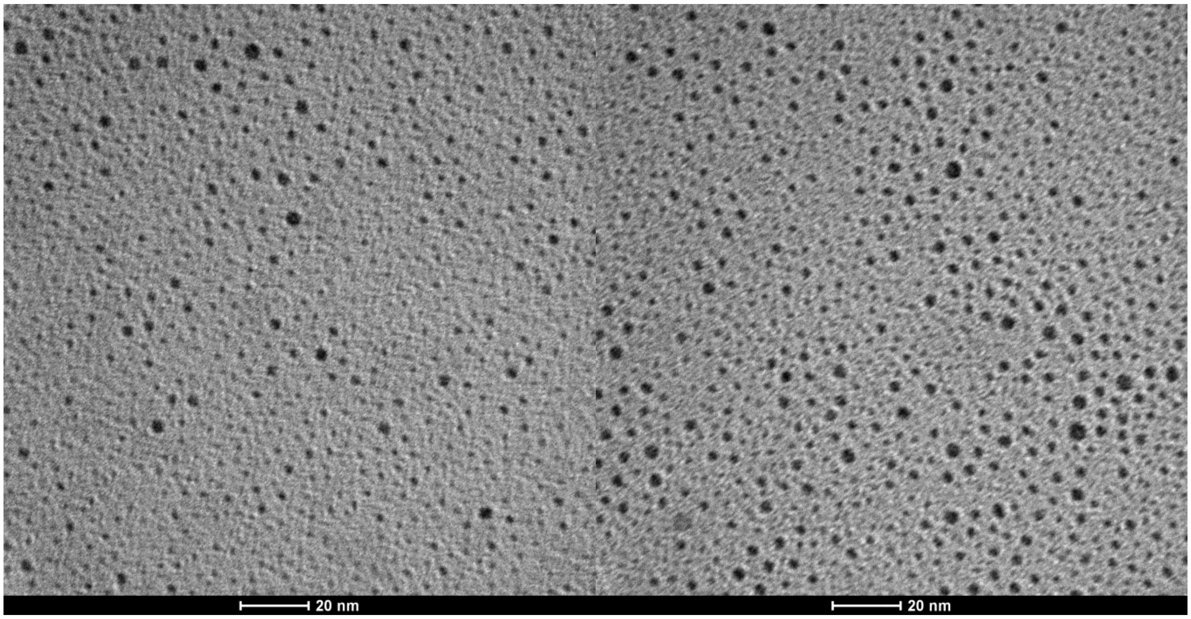

Figure S5. TEM micrographs of AuNP-**3**.

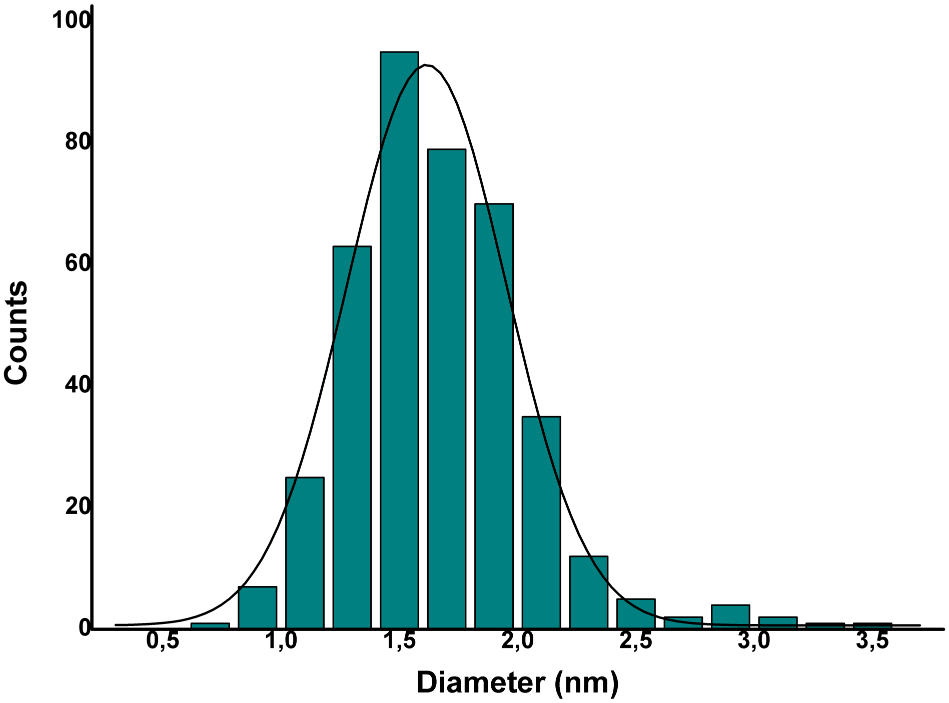

Figure S6. Size distribution of AuNP-**3** (d = 1.6 nm, σ = 0.4 nm).

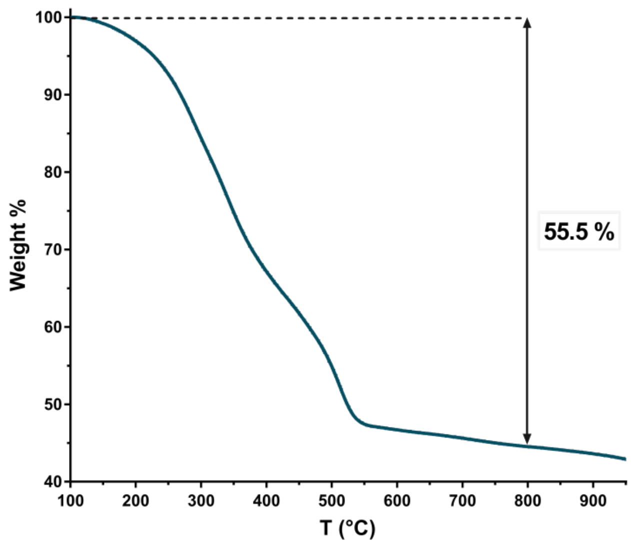

Figure S7. TGA analysis of AuNP-**3** (ramp 10 °C/min from 100 to 1000 °C, 0.5 mg of AuNPs, air atmosphere). From the combination of TEM and TGA data the average formula for AuNP-**3** is $Au_{127}SR_{65}$ with a thiol footprint of 0.16 nm^2.

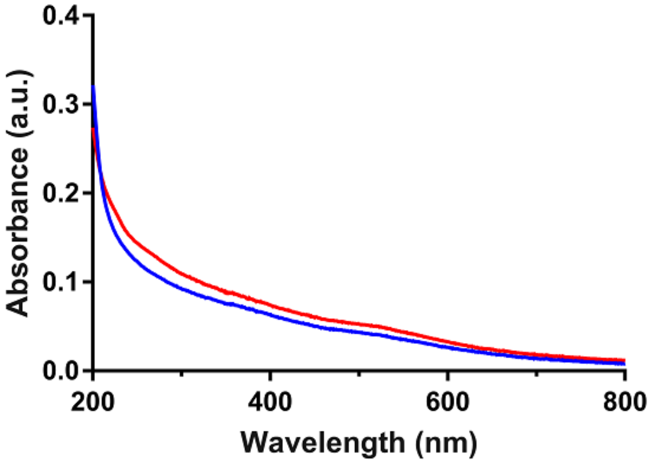

Figure S8. UV-Vis absorbance spectra of AuNP-**2** (blue) and AuNP-**3** (red) at 0.1 mg/mL concentration.

1.1.[13]C T_1 relaxation times, related to Figure 2

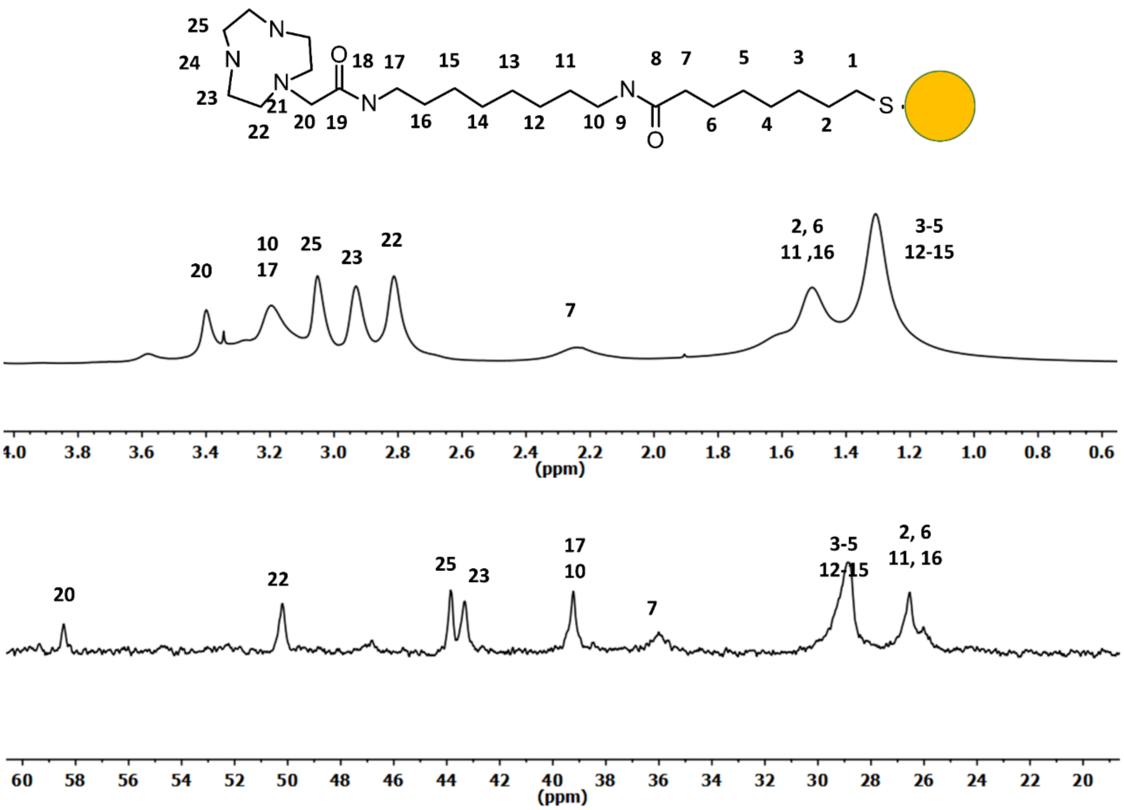

Figure S9. Structure, ^{1}H and ^{13}C NMR spectra of AuNP-**2** with signal assignation.

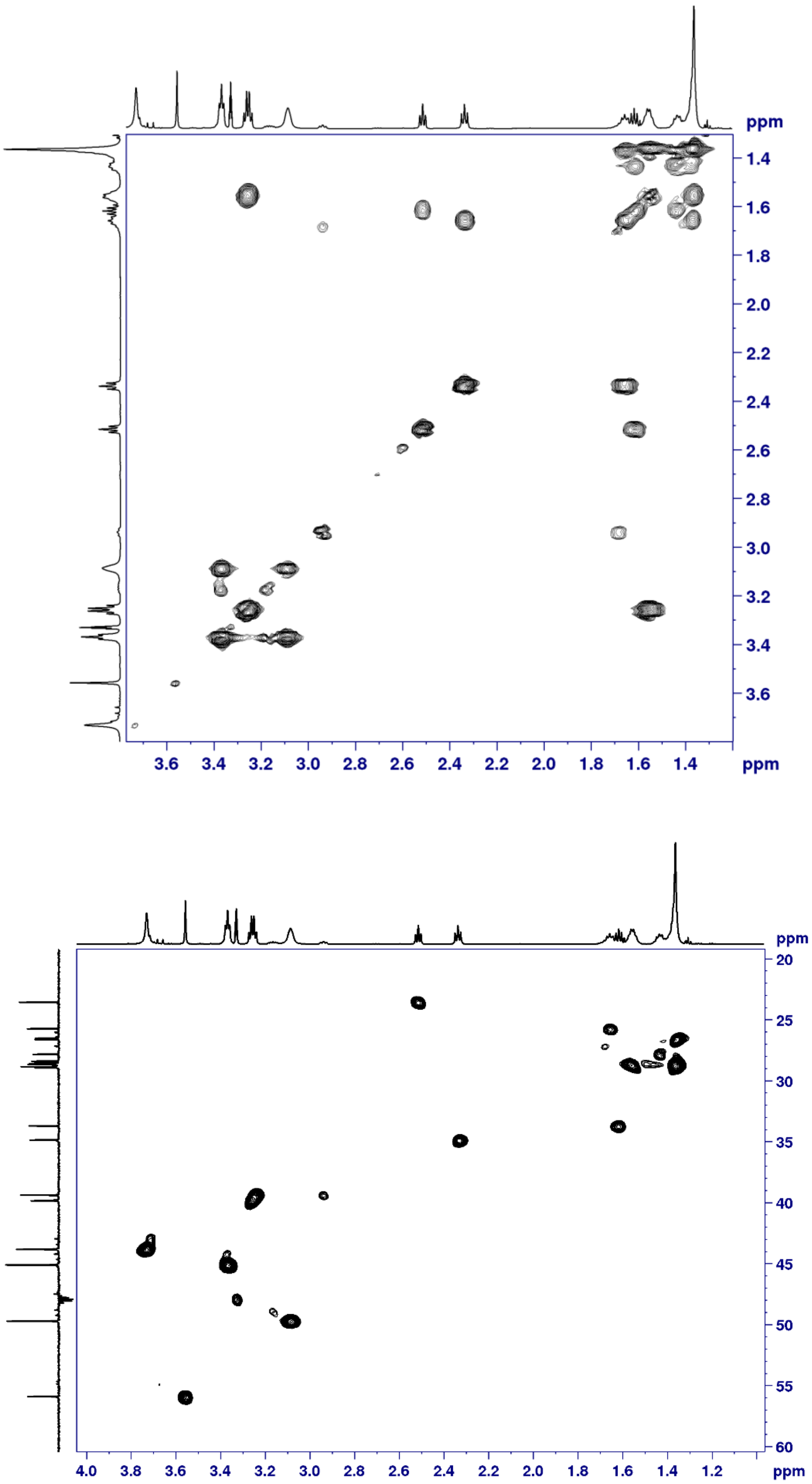

Figure S10. ^{1}H-^{1}H COSY (top) and ^{1}H-^{13}C HSQC spectra of the thiol **2** used for the signal assignation.

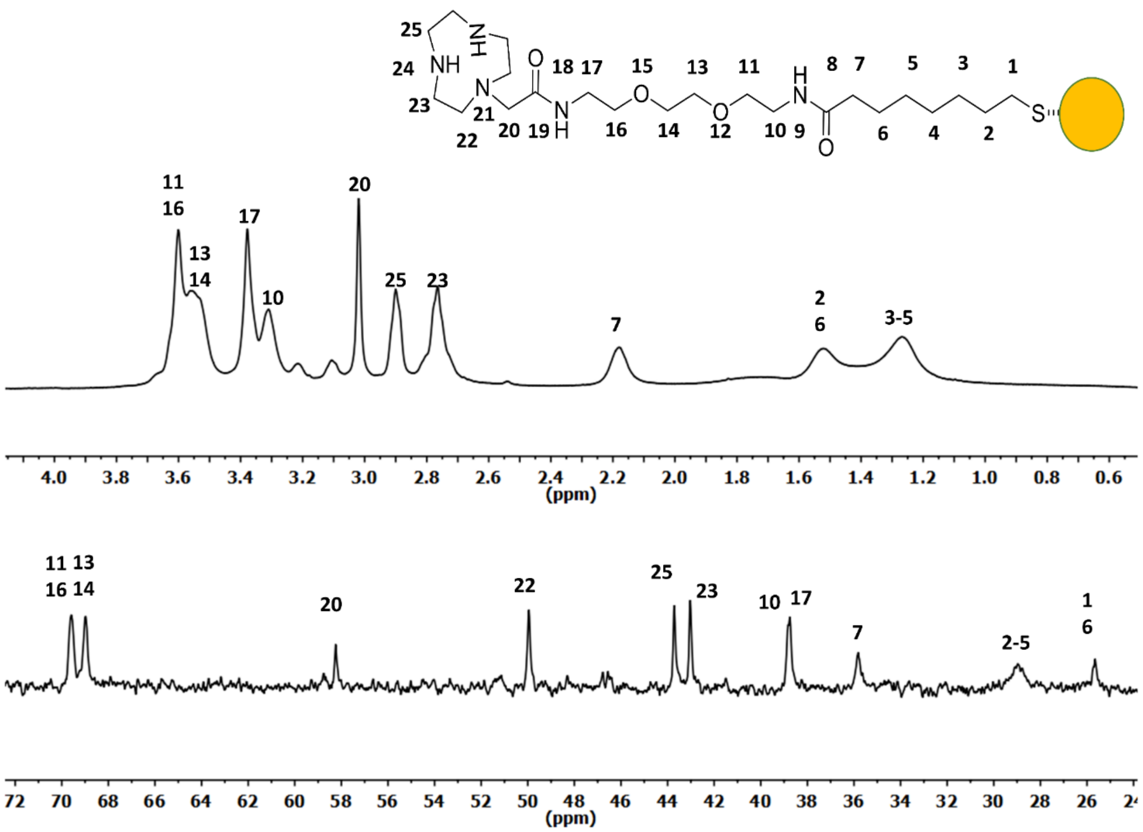

Figure S11. Structure, ^{1}H and ^{13}C NMR spectra of AuNP-**3** with signal assignation.

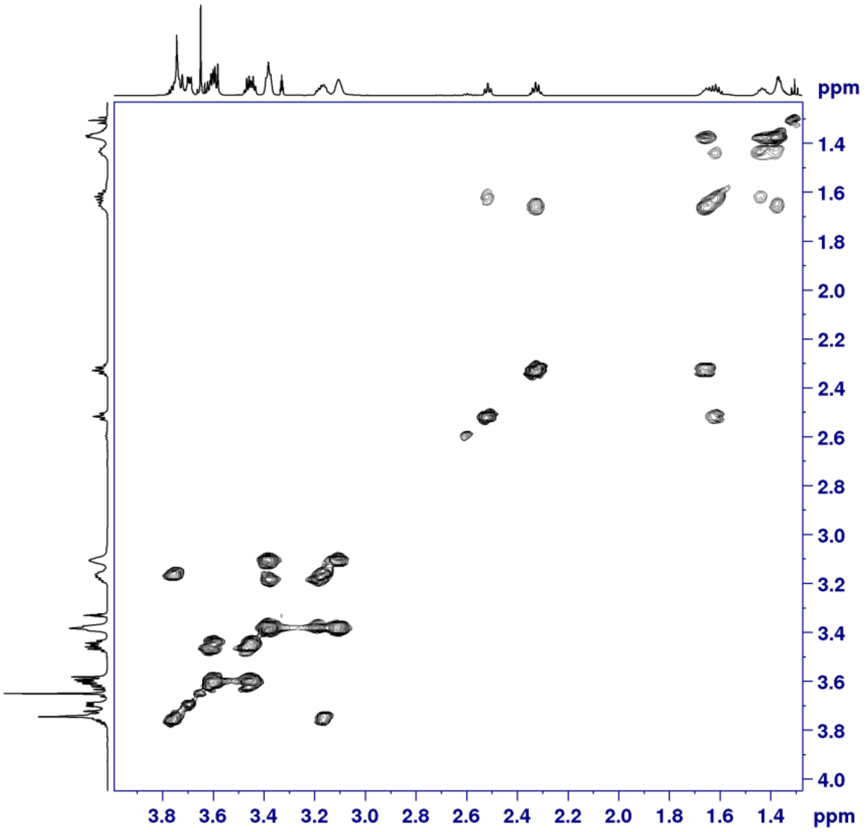

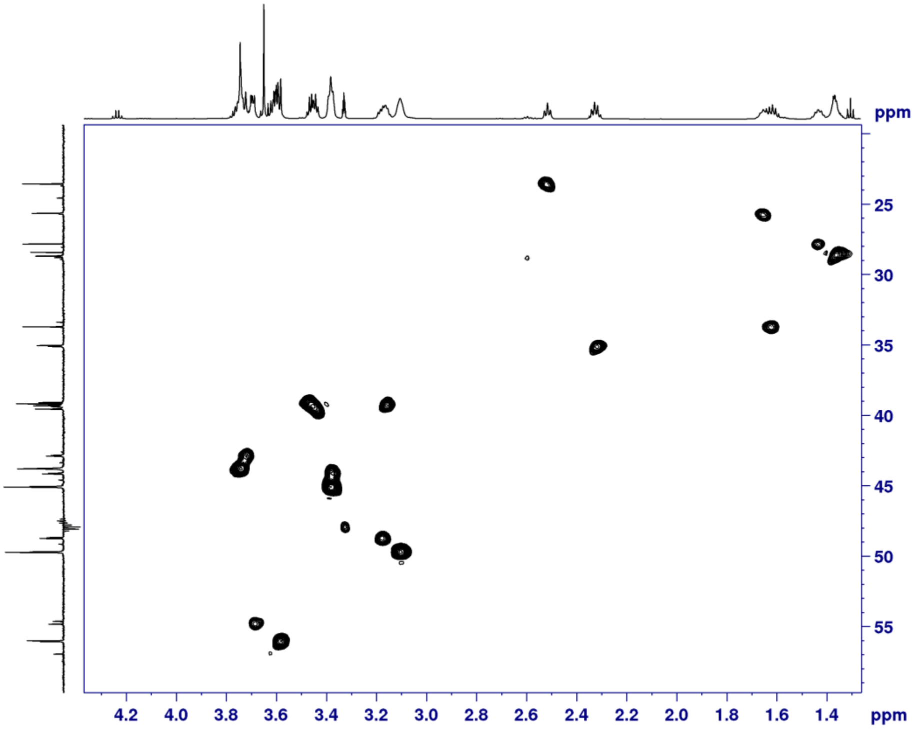

Figure S12. ^{1}H-^{1}H COSY (top) and ^{1}H-^{13}C HSQC spectra of the thiol **3** used for the signal assignation.

1.2. Autocorrelation functions of CH bonds, related to Figure 2

Rotational autocorrelation functions (RCFs) of CH bonds were calculated for each CH vector along the coating ligand chain (Figure S13A). Gromacs "gmx rotacf" tool was used for the calculation of the Fourier transform *C(t)* of RCFs, i.e. using the second order Legendre polynomial of the CH vectors:

$$C(t) = \langle P_2(\vec{\mu}(0) \cdot \vec{\mu}(t)) \rangle$$

where $\vec{\mu}$ is the CH unit vector and *P2* is the second Legendre polynomial and $\langle \dots \rangle$ is the time average. For our calculations, we considered snapshots between 25-200 ns with a time step of 2 ps, without removing the overall tumbling (Figure S13B,C and F) and after removing tumbling (Figure S13D,E andG). All Figures show only one of the hydrogens bound to C atoms, as the equivalent hydrogens (bound to the same C atom) give the same RCFs.

A Structure of the coating ligand of AuNP-**2** and AuNP-**3**. Carbon atoms of the inner thiol, of the outer linker and of the TACN unit are shown in blue, red and gray, respectively (matching all graphs below).

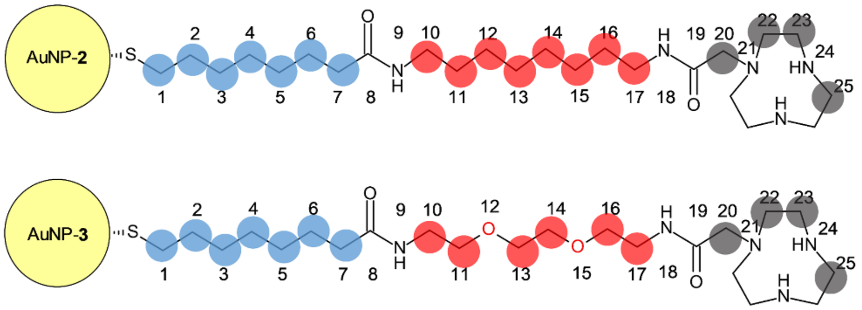

B Four replicas of 200ns-long MD simulation of AuNP-**2** (without removing overall tumbling)

AuNP-**2** replica 1 AuNP-**2** replica 2

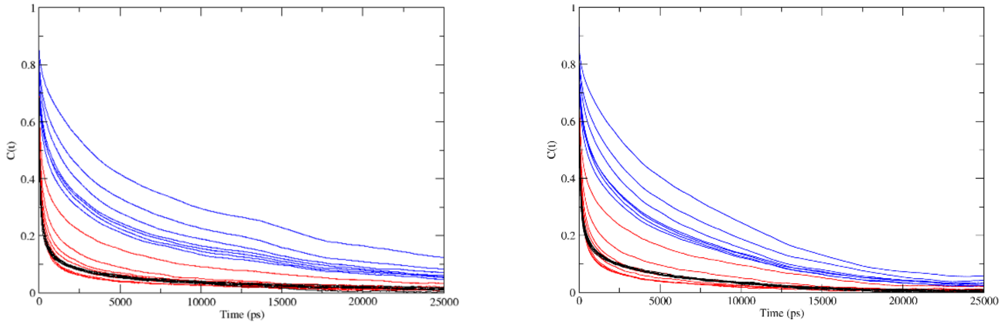

AuNP-**2** replica 3

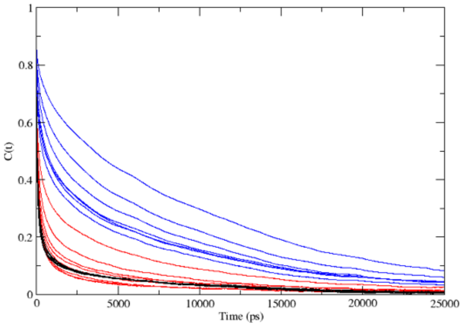

AuNP-**2** replica 4

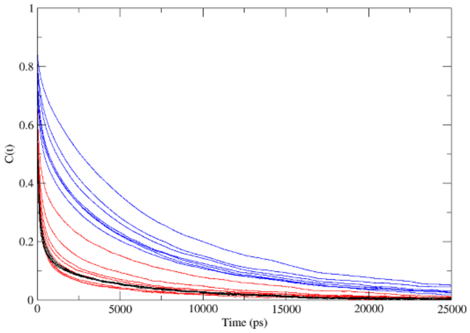

C Four replicas of 200ns-long MD simulation of AuNP-**3** (without removing overall tumbling)

AuNP-**3** replica 1

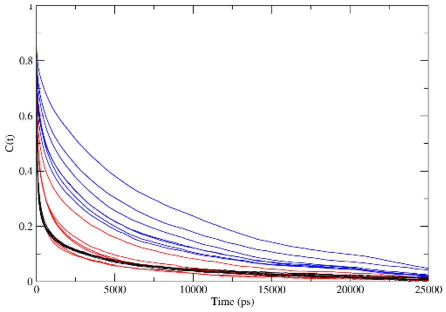

AuNP-**3** replica 2

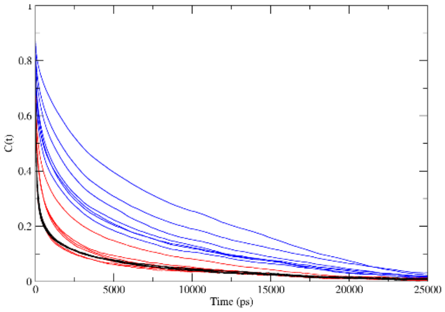

AuNP-**3** replica 3

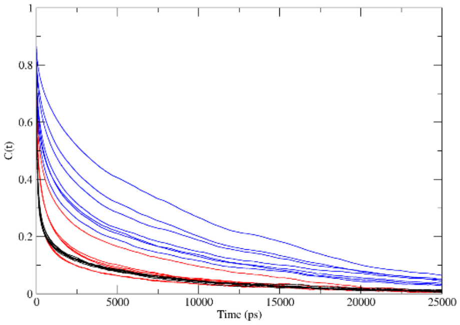

AuNP-**3** replica 4

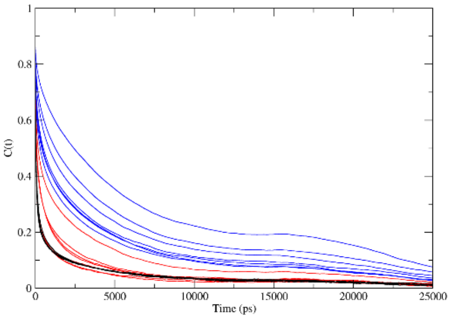

D Four replicas of 200ns-long MD simulation of AuNP-**2** (after removing overall tumbling)

AuNP-**2** replica 1

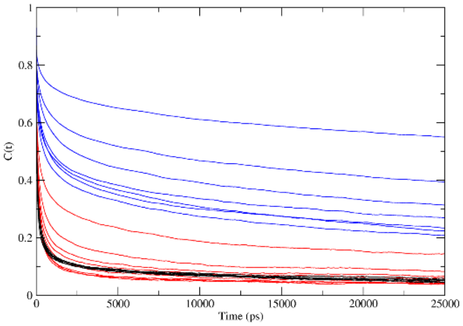

AuNP-**2** replica 2

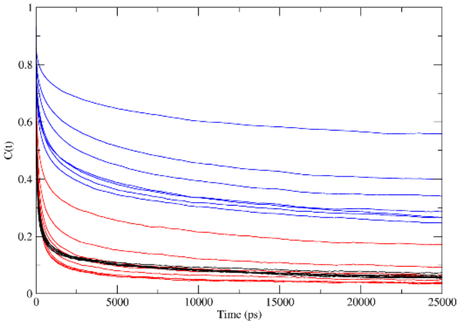

AuNP-**2** replica 3

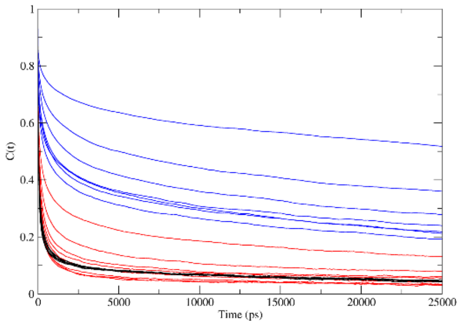

AuNP-**2** replica 4

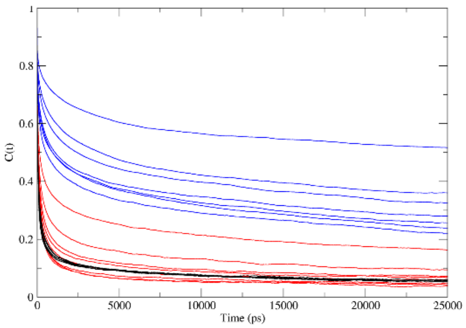

E Four replicas of 200ns-long MD simulation of AuNP-**3** (after removing overall tumbling)

AuNP-**3** replica 1

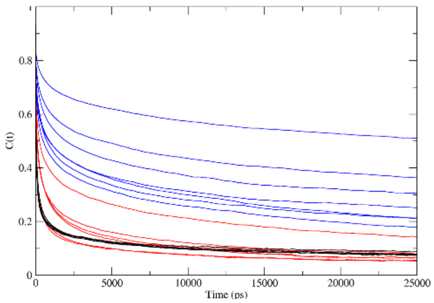

AuNP-**3** replica 2

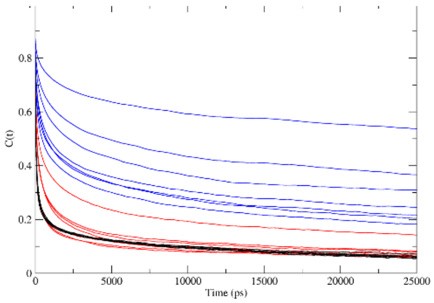

AuNP-**3** replica 3

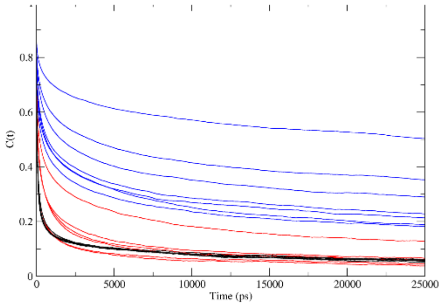

AuNP-**3** replica 4

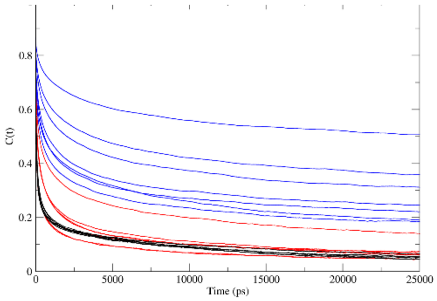

F Comparison of 200ns-long MD simulations of AuNP-**2** and AuNP-3 (without Zn atoms, without removing overall tumbling)

AuNP-**2** (noZn)

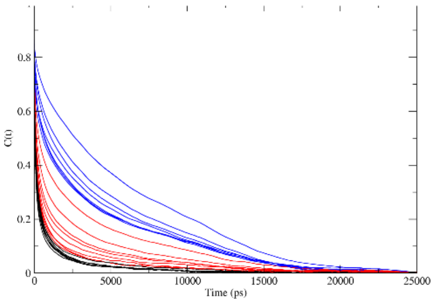

AuNP-**3** (noZn)

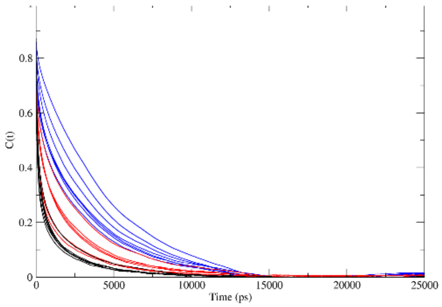

G Comparison of 200ns-long MD simulations of AuNP-**2** and AuNP-**3** without Zn atoms (after removing overall tumbling)

AuNP-**2** (noZn) AuNP-**3** (noZn)

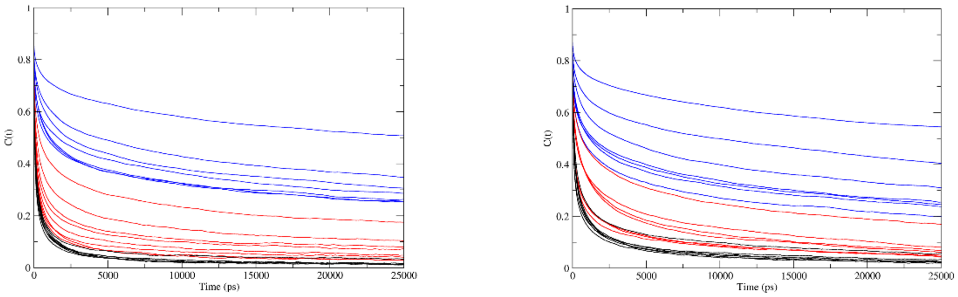

Figure S13. (A) Structure of coating ligand of AuNP-**2** and AuNP-**3** with the color-coding of the CH vectors matching the colour of the RCF decays in (**B-G**). Computed decays of rotational autocorrelation functions (RCFs) in AuNP-**2** and AuNP-**3**, derived from 200 ns MD simulations of AuNPs with the presence of Zn atoms (**B-E**) and without Zn atoms (**F, G**). Decays without removing overall tumbling (**B, C, F**) indicate an increased degree of mobility moving from the atoms closest to the gold core (in blue) toward the terminal part of the thiol (in red and black). Decays after removing overall tumbling (**D, E, G**) were used for calculations of T_1 relaxation times of individual CH vectors.

2. MD-derived characteristics of the coating ligands

Table S1. Computed and NMR-estimated T_1 relaxation times (in seconds) for ^{13}C nuclei of coating ligands of AuNP-2 and AuNP-3. For MD simulations we considered either the simulations without Zn or with Zn ions. Related to Figure 2.

	**AuNP-2**			**AuNP-3**		
Atom no.	**EXP no Zn**	**MD no Zn**	**MD Zn**	**EXP no Zn**	**MD no Zn**	**MD Zn**
**5**				0.164	0.205	0.244
**7**	0.241	0.237	0.269	0.211	0.215	0.241
**10**	0.218	0.237	0.272	0.194	0.215	0.247
**11**	0.250	0.234	0.263	0.251	0.228	0.239
**12**	0.250	0.232	0.257			
**13**	0.250	0.240	0.262	0.214	0.212	0.241
**14**	0.250	0.243	0.264	0.214	0.213	0.238
**16**	0.312	0.251	0.262	0.251	0.266	0.242
**17**	0.218	0.248	0.265	0.233	0.249	0.244
**20**	0.211	0.233	0.267	0.333	0.199	0.243
**22**	0.176	0.233	0.266	0.247	0.218	0.241
**23**	0.194	0.247	0.261	0.251	0.230	0.247
**25**	0.196	0.284	0.262	0.274	0.261	0.243

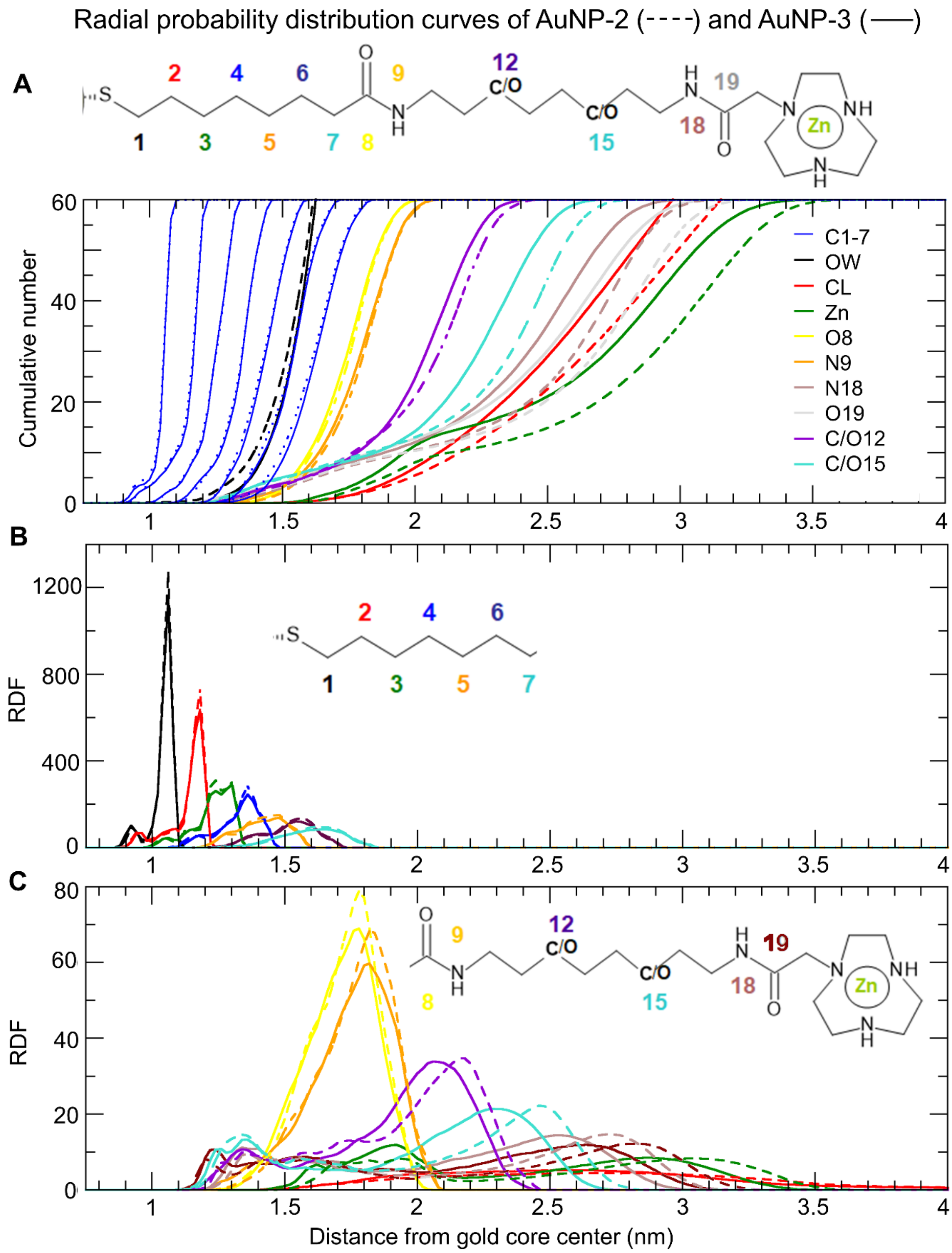

Figure S14. (a larger version of Figure 3C for better detail). (A) Cumulative number RDFs of selected atoms from both nanozymes (AuNP-2 in dashed lines and AuNP-3 in solid lines) together with Cl ions and the oxygen atom of water molecules (H_2O). (B) RDF functions of the first 7 carbons of the hydrophobic alkyl chains of AuNP-2 and AuNP-3. (C) RDF functions of the hydrophobic alkyl linker of AuNP-2 and hydrophilic PEG linker of AuNP-3 together with Zn ions chelated by TACN units and Cl ions (in red).

Table S2. Averaged number of contacts and hydrogen bonds between the coating ligands and water molecules (wat). Related to Figure 4.

**Contacts**	AuNP-**2**	AuNP-**3**
Zn … wat (<0.20 nm)	18.1 ± 4.8	15.8 ± 3.3
Zn … wat (<0.25 nm)	31.8 ± 8.9	27.9 ± 5.6
Zn … wat (<0.5 nm)	441.7 ± 49.0	407.0 ± 18.1
Zn … wat (<1.0 nm)	6464.6 ± 233.1	6264.4 ± 94.8
Zn … Cl (<0.25 nm)	31.6 ± 3.8	29.9 ± 2.9
Zn…O8=C (<0.25 nm)	7.4 ± 1.3	11.1 ± 2.1
Zn…O19=C (<0.25 nm)	0.2 ± 0.4	0.2 ± 0.4
**Hydrogen Bonds**		
C=O8...H-N9 (inner...inner)	20.5 ± 2.6	15.7 ± 2.5
C=O8...H-N18 (inner...outer)	0.6 ± 0.7	0.9 ± 0.8
C=O19...H-N9 (outer...inner)	0.1 ± 0.2	0.3 ± 0.5
C=O19...H-N18 (outer...outer)	0.4 ± 0.6	0.4 ± 0.6
O12...H-N9 (inner...inner)	-	5.3 ± 2.2
O12...H-N18 (inner...outer)	-	0.7 ± 0.9
O15...H-N9 (outer...inner)	-	0.7 ± 0.8
O15...H-N18 (outer...outer)	-	4.3 ± 2.0
C=O19...wat	36.0 ± 4.7	32.0 ± 4.3
N18-H...wat	50.2 ± 4.0	49.3 ± 3.3
C15/O15...wat	-	11.0 ± 3.0
C12/O12...wat	-	20.5 ± 3.8

N9-H…wat	39.2 ± 4.0	38.9 ± 3.7
C=O8…wat	86.1 ± 6.4	81.4 ± 7.2
O18, N19-H...wat	86.3 ± 6.7	81.3 ± 5.6
O8, N9-H…wat	125.3 ± 9.7	120.3 ± 9.2
total HBs of thiols with waters	323.1 ± 17.3	339.1 ± 13.9
SASA (nm^2)	248.5 ± 5.9	240 ± 5.6

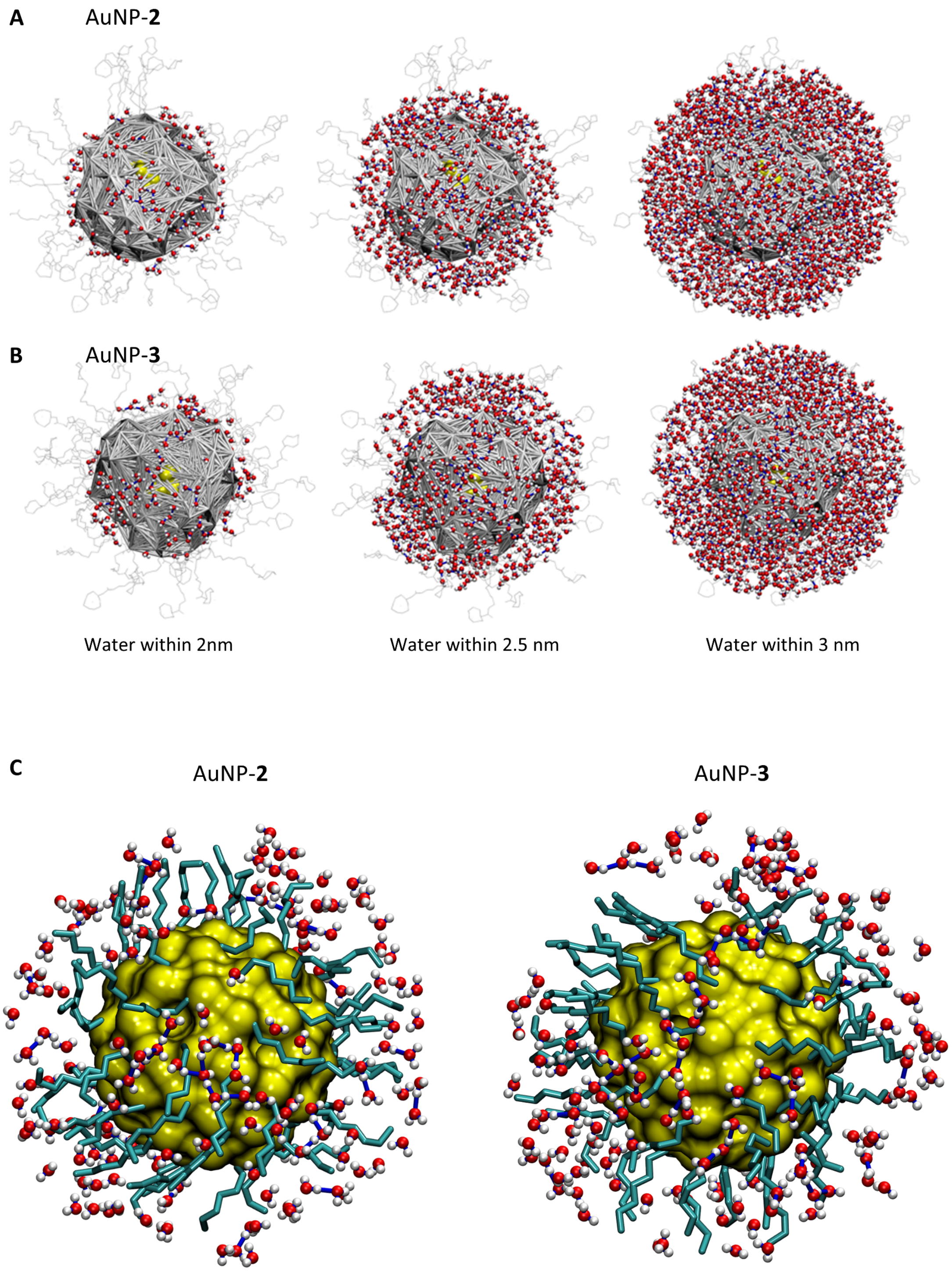

Figure S15. Representative snapshots of the AuNP-2 (A) and AuNP-3 (B) and solvent molecules at the distance of 2 nm, 2.5 nm and 3 nm from the center of mass of the gold core. Wires connect the first 7 carbon atoms of the hydrophobic alkyl part which are closer than 0.8 nm. (C) Zoom on hydrogen bond network formed on surface of the first 7 carbons of each nanozyme.

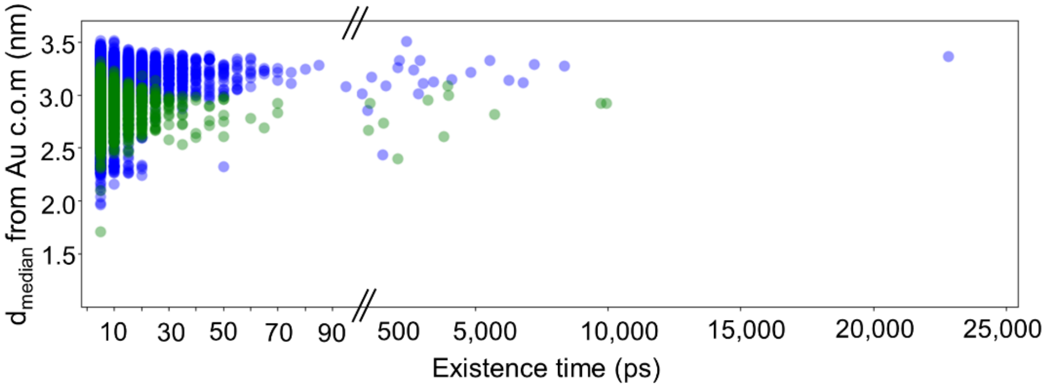

Figure S16. The formation of bimetallic binding sites in AuNP-**2** (in blue) and AuNP-**3** (in green) defined by a close proximity of two Zn ions (within 0.50 nm) showed in the graph of the residence time of each event and its median distance from the gold core (measured from the center of mass of involved Zn ions).

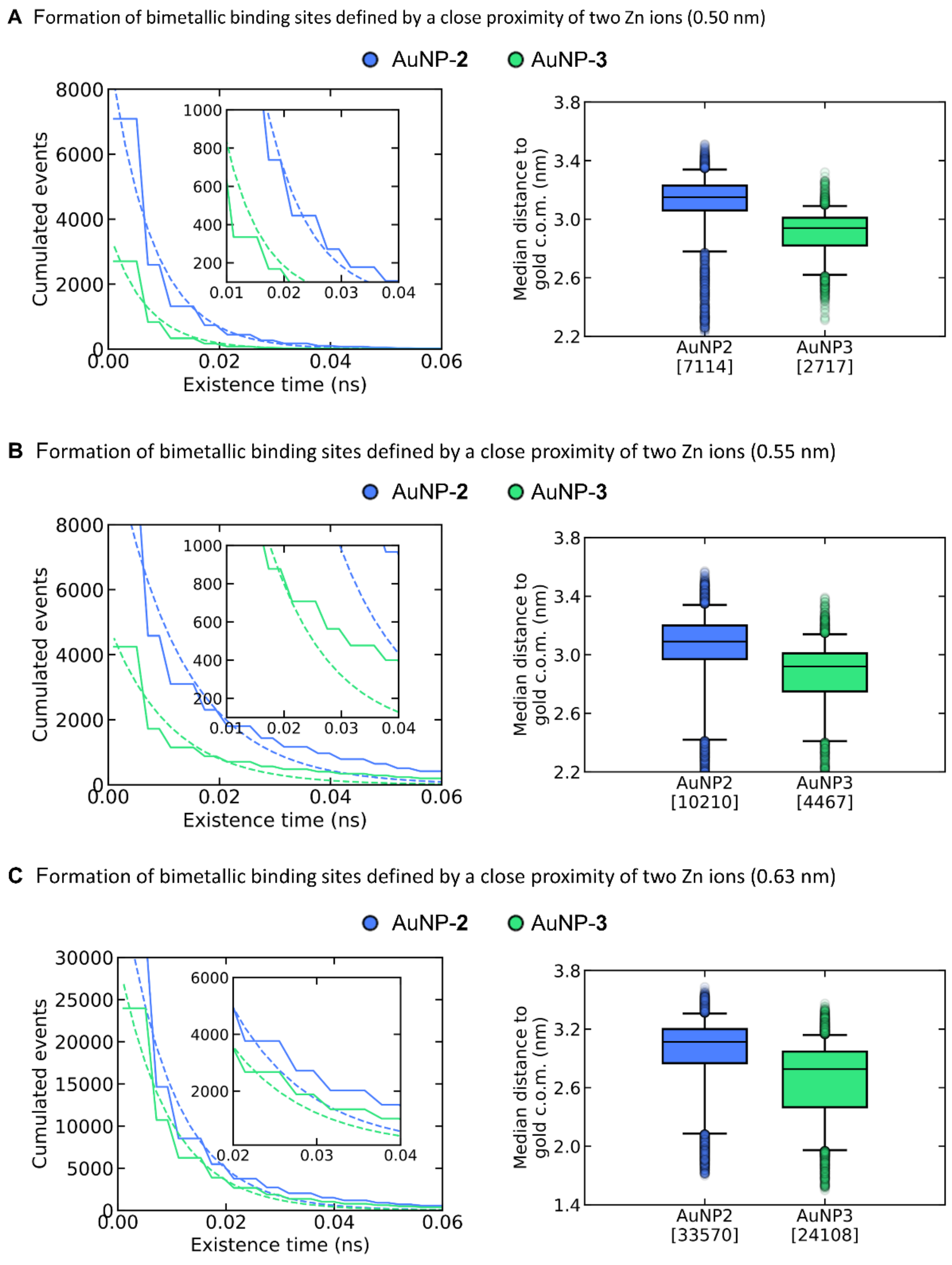

Figure S17. The formation of bimetallic binding sites in AuNP-**2** (in blue) and AuNP-**3** (in green), defined by a close proximity of two Zn atoms, using less tight thresholds than 0.5 nm (**A**; and Figure 5A), i.e., 0.55 nm in (**B**) and 0.63 nm in (**C**). The graphs (on the left) show the cumulated number of events at different existence times. The data (solid lines) were fitted to a single exponential (dotted line) to compare the decay rate (λ) of AuNP-**2** and AuNP-**3**, i.e. λ = 131.0 ns^{-1} and 150.4 ns^{-1}, respectively in (**A**); 81.2 ns^{-1} and 91.3 ns^{-1}, respectively in (**B**) and 106.6 ns^{-1} and 106.9 ns^{-1}, respectively in (**C**). The boxplots (on the right) show the distributions of the median distance of bimetallic sites from the center of the gold core. Boxes extend from the 25th to the 75th quantiles, and the whiskers go from the 5th to the 95th quantiles, with a median (50th quantile) as a black line. The square brackets on the x-axis show the total number of binding sites for each nanoparticle.

3. MD-derived characteristics of the coating ligands in AuNP-2 and AuNP-3 in the presence of the substrate.

Table S3. The difference of measures estimated on AuNP-2 and AuNP-3 with and without the presence of substrate molecules and at room temperature T=25 °C or at temperature of kinetic measurements T=40 °C. All values were calculated on a joined trajectory from all replicas. Analysis was done each 10 ps, discarding the first 25 ns of simulation as an equilibration. Root-mean-squared displacement (RMSD) values are measured from the fully equilibrated structure (200 ns, i.e., in t=0 ns of production run). Interactions were quantified by "gmx hbonds contact" tool using threshold distances. Hydrogen bonds were quantified by "gmx hbonds" tool. Atoms of the "inner" and "outer" amide groups forming inter-ligand hydrogen bonds are marked accordingly.

Systems / Measures	AuNP-**2** alone T=25 °C	AuNP-**3** alone T=25 °C	AuNP-**2**/sub T=25 °C	AuNP-**3**/sub T=25 °C	AuNP-**2**/sub T=40 °C	AuNP-**3**/sub T=40 °C
RMSD (nm)	1.30 ± 0.05	1.18 ± 0.05	1.28 ± 0.06	1.22 ± 0.07	1.40 ± 0.22	1.05 ± 0.08
Rg (nm)	1.73 ± 0.02	1.65 ± 0.01	1.71 ± 0.01	1.64 ± 0.01	1.71 ± 0.02	1.63 ± 0.01
Eccentricity	0.055 ± 0.005	0.050 ± 0.003	0.054 ± 0.004	0.048 ± 0.004	0.068 ± 0.001	0.049 ± 0.005
SASA (nm^2)	248.5 ± 5.9	240 ± 5.6	254.0 ± 6.0	243.7 ± 5.7	246.5 ± 6.3	235.6 ± 5.5
**INTERACTIONS**						
Zn…wat (<0.20 nm)	18.1 ± 4.8	15.8 ± 3.3	16.8 ± 3.5	14.3 ± 3.0	12.1 ± 2.8	10.5 ± 3.4
Zn…wat (<0.25 nm)	31.8 ± 8.9	27.9 ± 5.6	29.8 ± 5.7	25.6 ± 4.9	20.2 ± 4.6	17.8 ± 5.1
Zn…wat (<0.50 nm)	441.7 ± 49.0	407.0 ± 18.1	408.7 ± 16.2	386.5 ± 15.3	367.7 ± 16.8	343.6 ± 14.5
Zn…wat (<1.00 nm)	6464.6 ± 233.1	6264.4 ± 94.8	6217.0 ± 90.1	6039.8 ± 87.7	5975.9 ± 93.0	5780.8 ± 84.9
Zn…Cl (<0.25 nm)	31.6 ± 3.8	29.9 ± 2.9	28.9 ± 3.7	28.1 ± 3.0	27.6 ± 3.3	24.8 ± 3.1
Zn…O8=C (<0.25 nm)	7.4 ± 1.3	11.1 ± 2.1	6.8 ± 1.9	10.0 ± 2.0	9.6 ± 1.3	15.3 ± 1.5
Zn…O19=C (<0.25 nm)	0.2 ± 0.4	0.2 ± 0.4	0.2 ± 0.4	0.2 ± 0.4	0.2 ± 0.4	0.2 ± 0.4
Zn…O12 (<0.25 nm)	-	0	-	0	-	0
Zn…O15 (<0.25 nm)	-	0	-	0	-	0
**Hydrogen Bonds**						
C=O8…H-N9 (inner…inner)	20.5 ± 2.6	15.7 ± 2.5	20.5 ± 2.5	15.5 ± 2.9	20.2 ± 2.9	17.2 ± 3.4

C=O8…H-N18 (inner…outer)	0.6 ± 0.7	0.9 ± 0.8	0.8 ± 0.8	0.3 ± 0.5	0.5 ± 0.7	0.9 ± 0.8
C=O19…H-N9 (outer…inner)	0.1 ± 0.2	0.3 ± 0.5	0.1 ± 0.3	0.4 ± 0.6	0.1 ± 0.3	0.6 ± 0.8
C=O19…H-N18 (outer…outer)	0.4 ± 0.6	0.4 ± 0.6	0.4 ± 0.6	1.1 ± 0.8	0.4 ± 0.6	0.3 ± 0.5
O12…H-N9 (inner…inner)	-	5.3 ± 2.2	-	4.9 ± 2.2	-	5.5 ± 2.0
O12…H-N18 (inner…outer)	-	0.7 ± 0.9	-	0.5 ± 0.7	-	0.6 ± 0.7
O15…H-N9 (outer…inner)	-	0.7 ± 0.8	-	0.6 ± 0.8	-	0.5 ± 0.7
O15…H-N18 (outer…outer)	-	4.3 ± 2.0	-	4.4 ± 2.0	-	3.7 ± 1.9
C=O19…wat	36.0 ± 4.7	32.0 ± 4.3	35.0 ± 4.6	30.6 ± 4.3	32.5 ± 4.6	27.8 ± 4.2
N18-H…wat	50.2 ± 4.0	49.3 ± 3.3	48.4 ± 3.4	48.0 ± 3.3	46.4 ± 3.4	46.7 ± 3.5
C15/O15…wat	0	11.0 ± 3.0	0	10.4 ± 2.9	0	9.3 ± 2.9
C12/O12…wat	0	20.5 ± 3.8	0	19.4 ± 3.8	0	16.6 ± 3.6
N9-H…wat	39.2 ± 4.0	38.9 ± 3.7	32.8 ± 3.5	33.4 ± 4.4	34.9 ± 3.3	32.3 ± 3.9
C=O8…wat	86.1 ± 6.4	81.4 ± 7.2	87.1 ± 8.2	81.3 ± 7.4	76.7 ± 5.6	68.2 ± 6.5

A

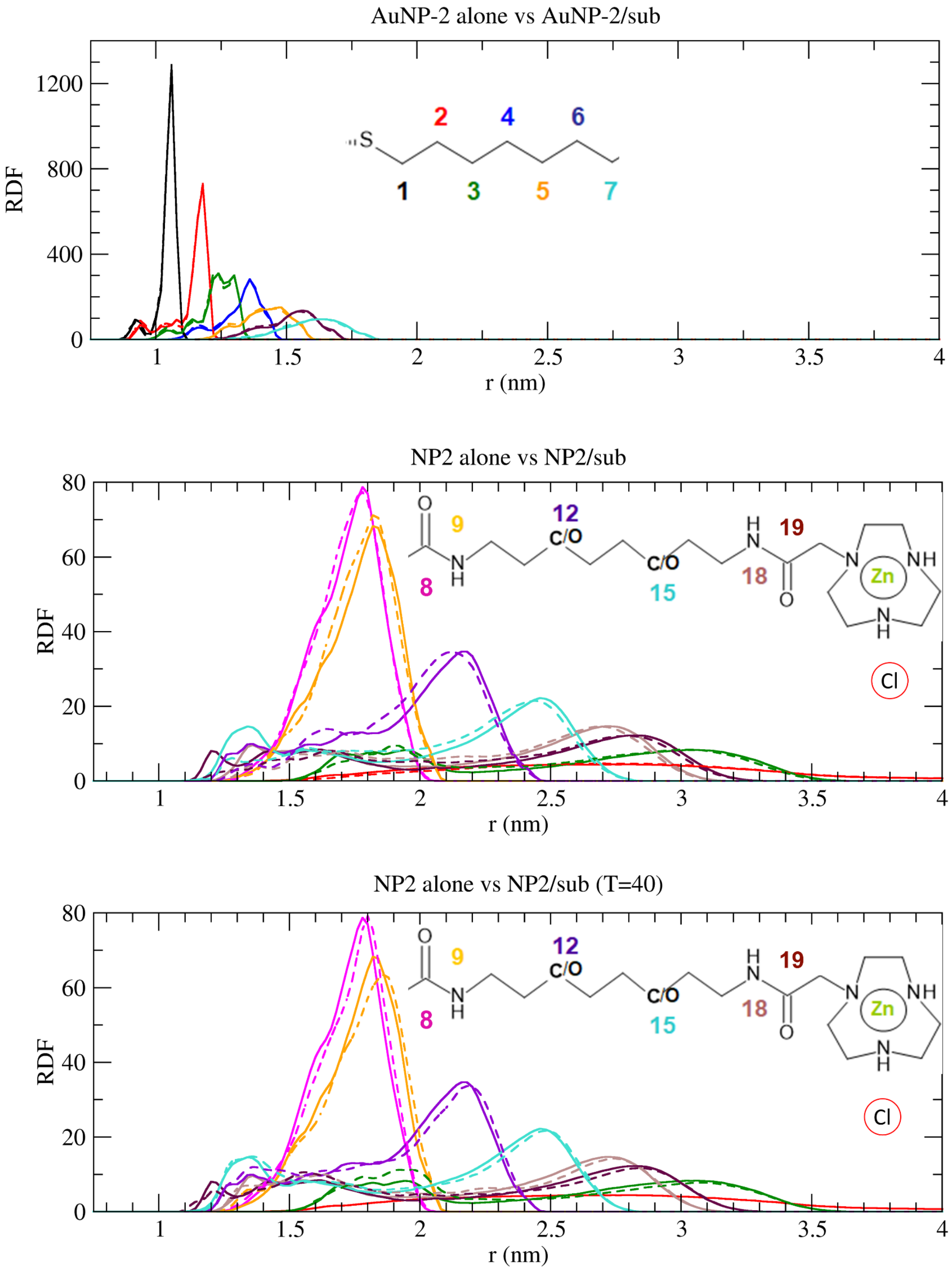
AuNP-2 alone vs AuNP-2/sub
RDF
r (nm)
1200
800
400
0
1
1.5
2
2.5
3
3.5
4
S
1
2
3
4
5
6
7
NP2 alone vs NP2/sub
RDF
r (nm)
80
60
40
20
0
O
N
H
8
9
12
C/O
C/O
15
H
N
18
19
O
N
NH
N
H
Zn
Cl
NP2 alone vs NP2/sub (T=40)

B

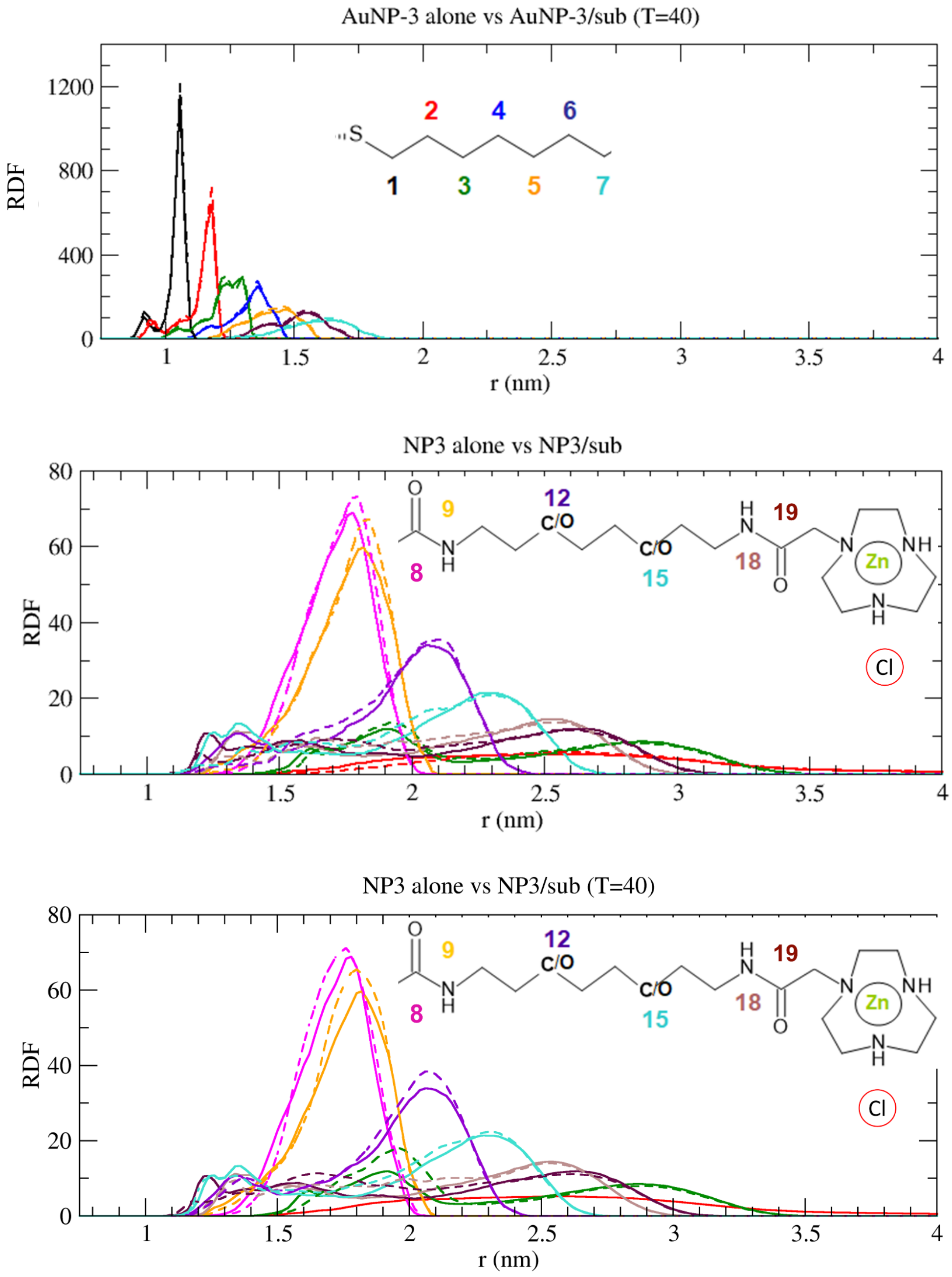

Figure S18. Comparison of radial distribution functions (RDFs) from different simulations of studied nanoparticles, with and without the presence of substrate and at different temperatures. **(A)** on top: RDFs of the first 7 carbons of the hydrophobic alkyl chains of AuNP-**2** from MD without substrate at 25 °C (dashed lines) and with substrate at 25°C (solid lines); and RDF functions of the hydrophobic alkyl chain of AuNP-**2** from simulations in the presence of substrate at 25 °C (in the middle) and from simulations with the substrate at 40 °C (at the bottom), in both cases compared with RDFs from MD without the substrate at 25°C (dashed lines). **(B)** the same comparison of RDFs from different simulations and individual parts of coating ligands of AuNP-**3**.

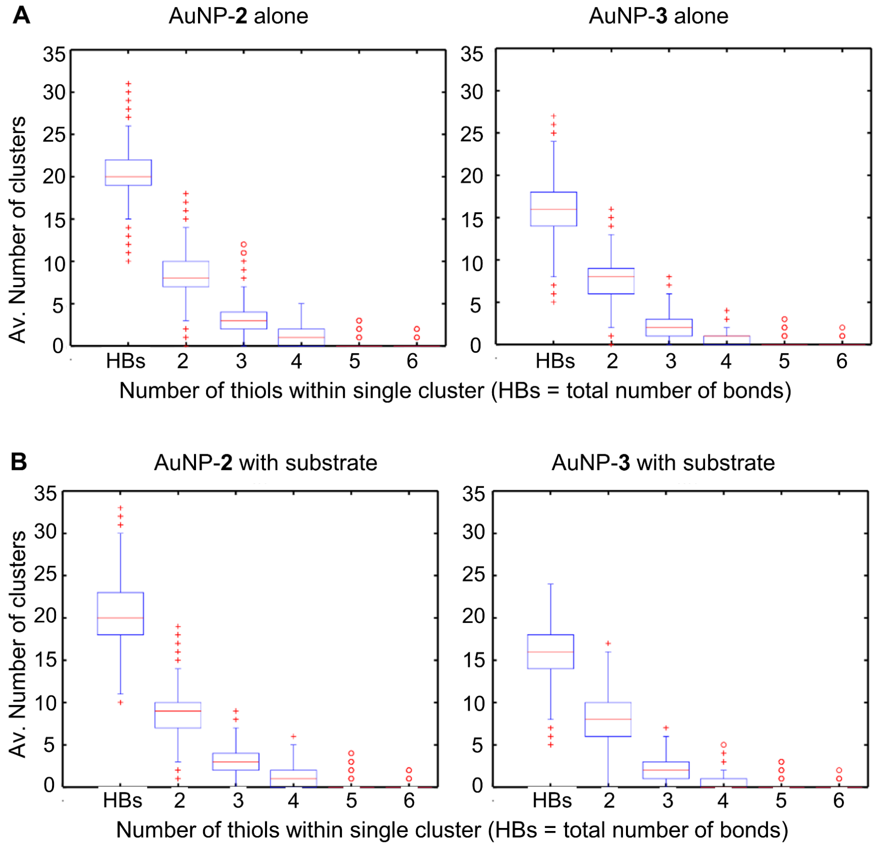

Figure S19. Comparison of average number of bundles formed on the surface of each nanoparticle (AuNP-**2**, left column and AuNP-**3**, right column) during MD simulations without and with the presence of substrate molecules.

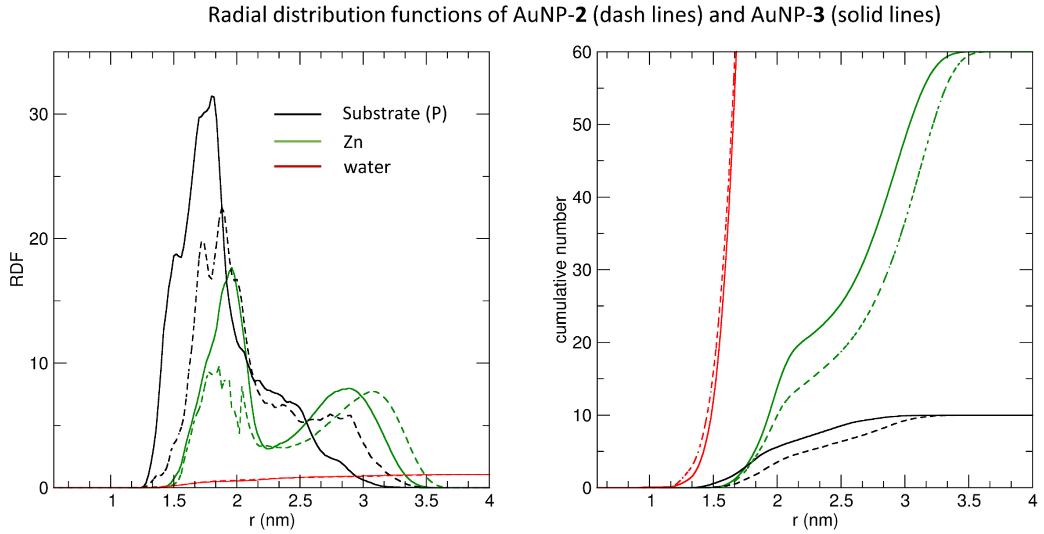

Figure S20. Comparison of radial distribution functions (RDFs; on left) and cumulative RDFs (on right) of P1 atoms of HPNP substrate (in black), Zn ions (in green) and Oxygen atom of water molecules (in red) in AuNP-**2** (dash lines) and AuNP-**3** (solid lines).

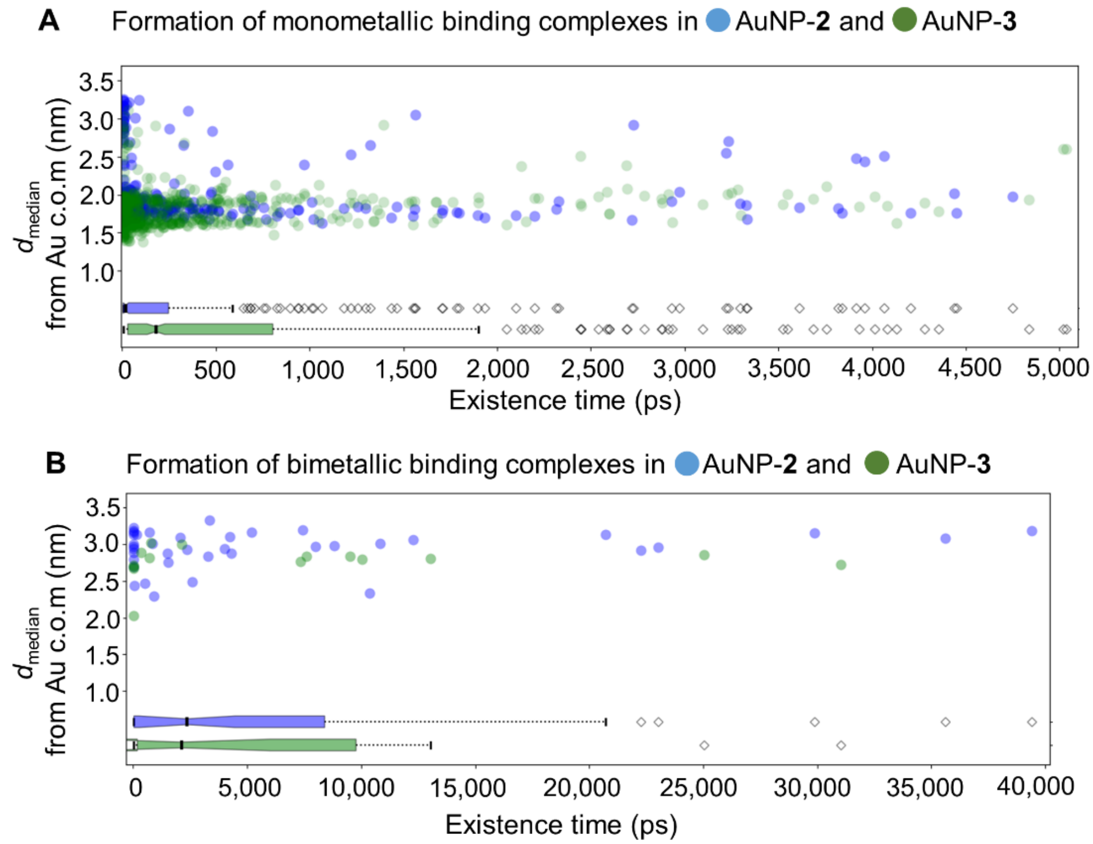

Figure S21. The formation of AuNP-Zn/substrate binding complexes. The graphs compare the formation of monometallic (**A**) and bimetallic (**B**) binding complexes in AuNP-**2** (in blue) and AuNP-**3** (in green), by showing the individual events plotted with their residence time against the median distance (of the central P atom) from the center of the gold core.

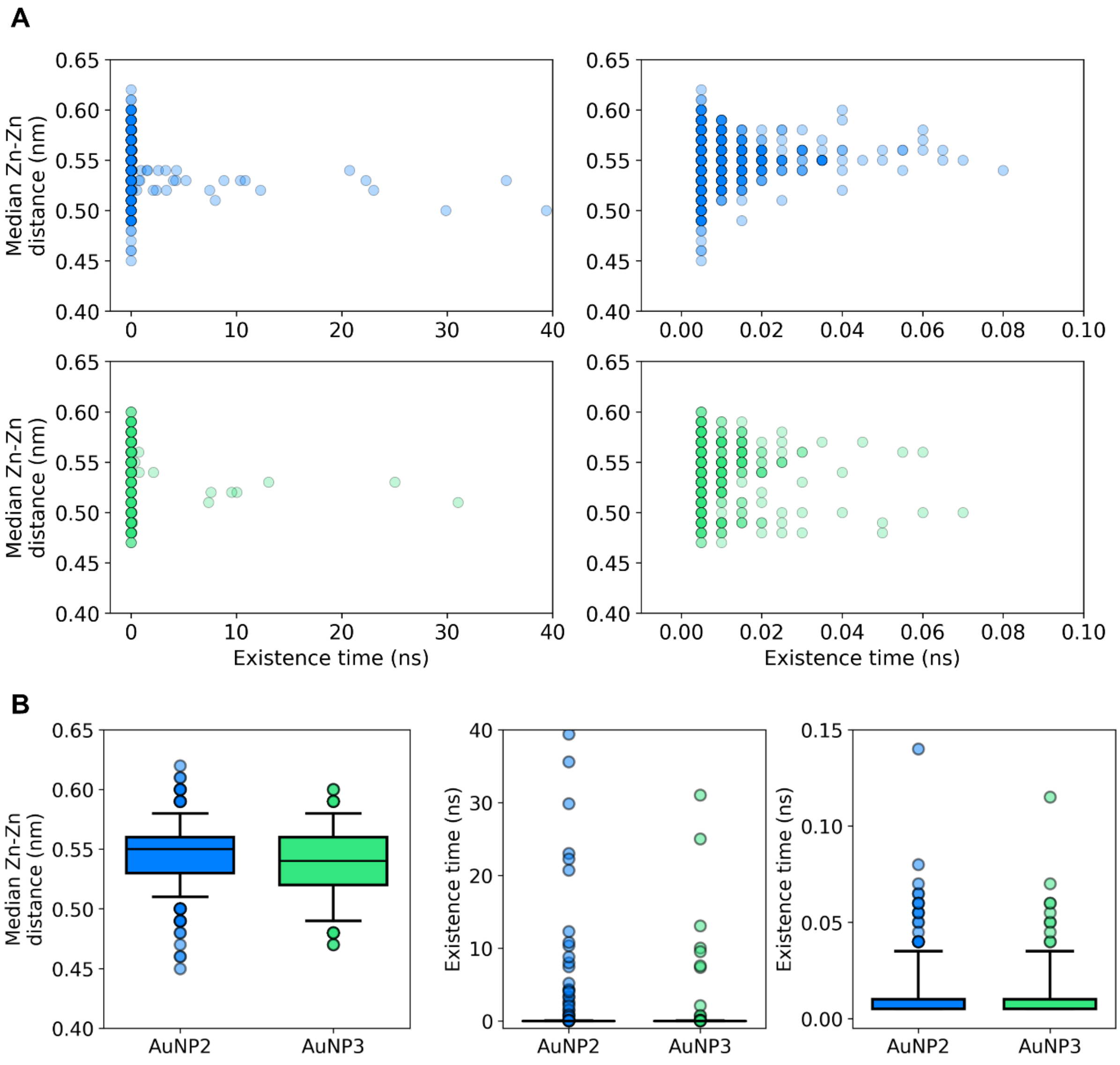

Figure S22. Inter-Zn distances in bimetallic binding complexes of AuNP-**2** (in blue) and AuNP-**3** (in green). (**A**) Median Zn-Zn distances of each bimetallic complex formation plotted with its existence time (ns); (**B**) The boxplots show the Zn-Zn distance and existence time distributions of bimetallic binding complexes.

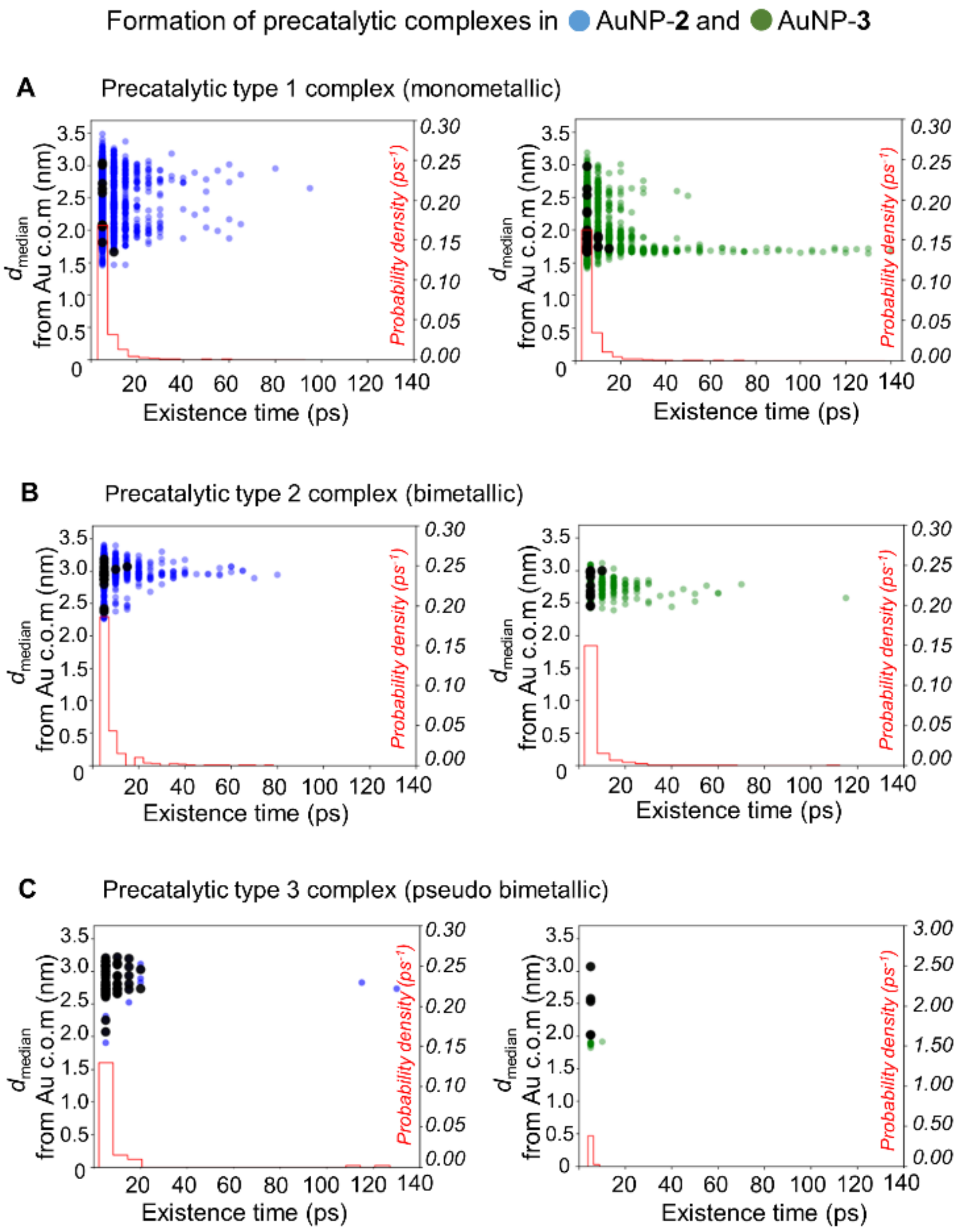

Figure S23. Formation of 3 different precatalytic complexes in AuNP-**2** (on left with blue dots) and AuNP-**3** (on right with green dots); monometallic precatalytic type 1 complex (**A**), bimetallic precatalytic type 2 complex (**B**) and pseudo bimetallic precatalytic type 3 complex (**C**). Each dot corresponds to the event of formation with its residence time and median distance of the central phosphorus from the gold core center of mass (d_{median}). Black dots represent the solvated states of precatalytic complexes with one Zn-coordinated water molecule. Probability density in ps^{-1} for each system is shown in a red color.

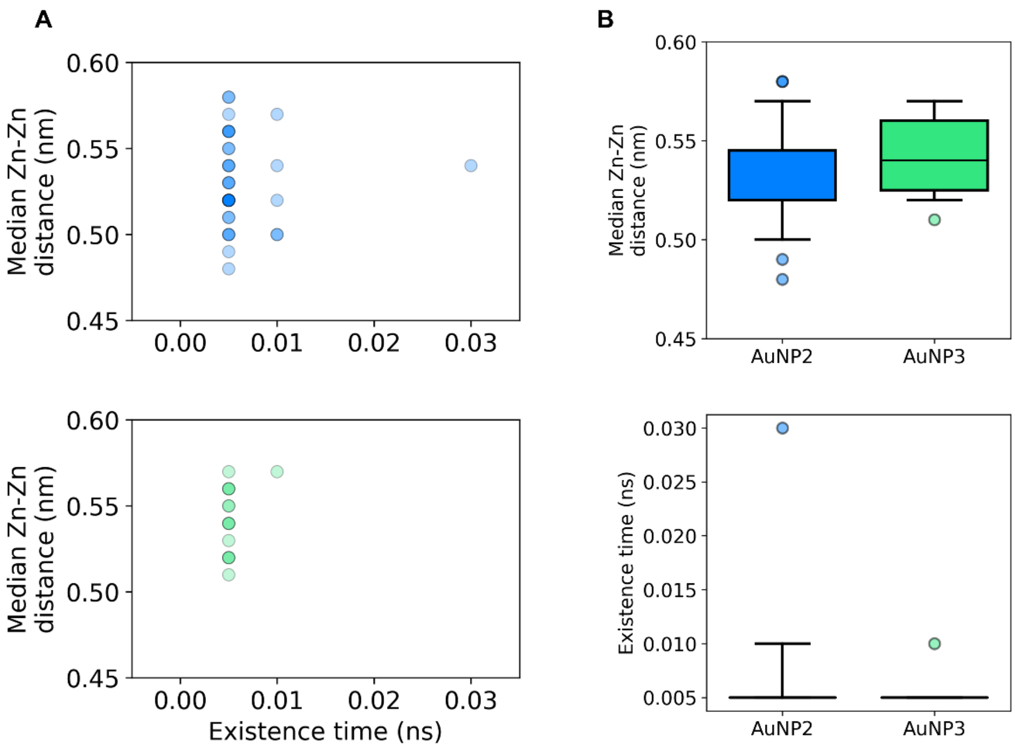

Figure S24. Inter-Zn distances in bimetallic type 2 precatalytic complexes of AuNP-**2** (in blue) and AuNP-**3** (in green). (**A**) Median Zn-Zn distances of each bimetallic complex formation plotted with its existence time (ns); (**B**) The boxplots show the Zn-Zn distance and existence time distributions.

3.1. Transitions between binding and precatalytic states

We tracked the binding of HPNP to AuNP-**2** or AuNP-**3**. We used the following definitions, which are the same used in the maintext with the exception of "monometallic" and "bimetallic" binding complexes being here more restrictive:

- Type 1 (monometallic): the distances d_1, d_2, and d_3 between Zn1 and both phosphate's oxygen atoms (O4, O5), together with hydroxyl's oxygen (O3) are below 0.25 nm. No other Zn ion is involved in HPNP binding.
- Type 2 (bimetallic): both oxygen atoms of the substrate's phosphate group (O4 and O5) are bound to two different Zn ions, simultaneously (d_1 and $d_2 < 0.25$ nm), while one of the Zn ions coordinates the oxygen of the HPNP's hydroxyl (O3 with d_3 within 0.25 nm).
- Type 3 (pseudo bimetallic): one of the Zn ions directly coordinates the oxygen atoms O4 and/or O5 of the phosphate group (with d1 and/or d2 within 0.25 nm), while the second Zn interacts only with O3 of the hydroxyl group (d3 within 0.25 nm).
- Monometallic: one or both oxygen atoms of the phosphate group (O4/O5) in HPNP coordinate(s) to a single Zn ion (d_1/d_2 within 0.25 nm), and no additional Zn is involved in HPNP binding. There are no other conditions defined, e.g. d_3 between Zn and O3 oxygen and thus the group of Monometallic complexes does not overlap with groups of Precatalytic type 1 and type 3 complexes.
- Bimetallic: each of two oxygen atoms of the phosphate group (O4/O5) in HPNP coordinates different Zn atoms (Zn1/Zn2) simultaneously. There are no other conditions defined, e.g. d_3

between Zn and O3 hydroxyl oxygen of HPNP. This group thus does not overlap with the group of Precatalytic type 2 complexes.

- Unbound: none of the above, i.e., the distance between HPNP and a Zn ion is higher than 2.5 nm

Using the above definition of the AuNP/substrate states, we calculated the binding complex for each HPNP molecule at each MD snapshot. For each trajectory and for each of the 10 HPNP molecules, we calculated a one-dimensional time series *n(t)* with *n* being the binding state at time *t*. To study the interconversion between the different states, we calculated from each *n(t)* the associated transition matrix $\{T_{mn}\}$. The forty (10 HPNP molecules and 4 MD simulations) $\{T_{mn}\}$ matrices were, then, added. The final transition matrix was row-normalized so that each T_{mn} corresponds to the probability that the state *n* transitions to state *m* within a certain lag time τ. In our case, we used τ = 5 ps, which is the time between the saved snapshots during our MD simulations. The diagonal elements of the matrix, i.e., T_{nn}, describe the metastability of the state *n*.

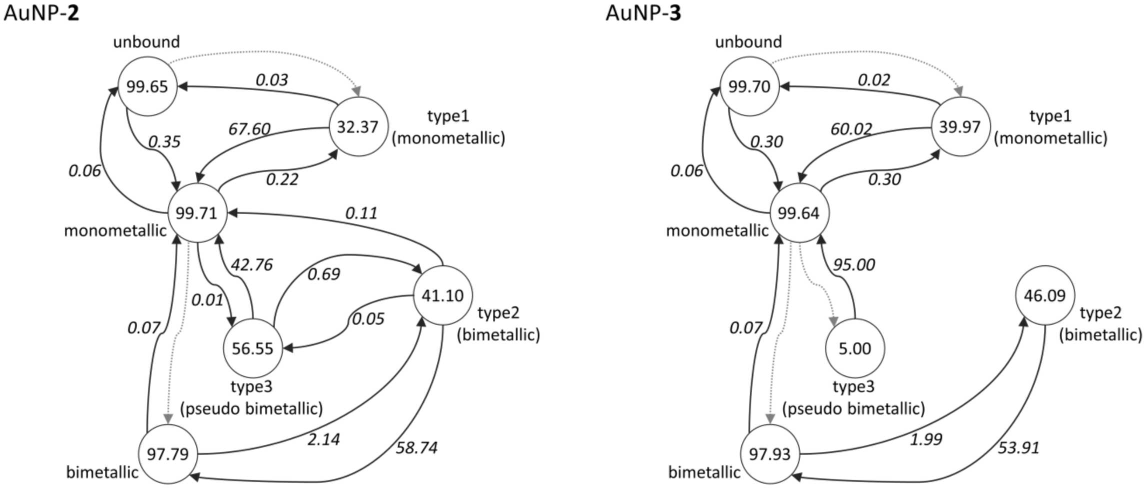

Figure S25. Representation of the transition matrix $\{T_{mn}\}$ between the different binding and precatalytic states. The nodes (i.e., the circles) are the considered states and the edges (i.e., the arrows) are the transitions between these states. The number in each node *n* is the metastability of that state, while the number on each edge from node *m* to node *n* is the transition probability T_{mn} of moving from state *m* to state *n* after a lag time of 5 ps. The light gray dotted edges have $T_{mn} < 0.01$.

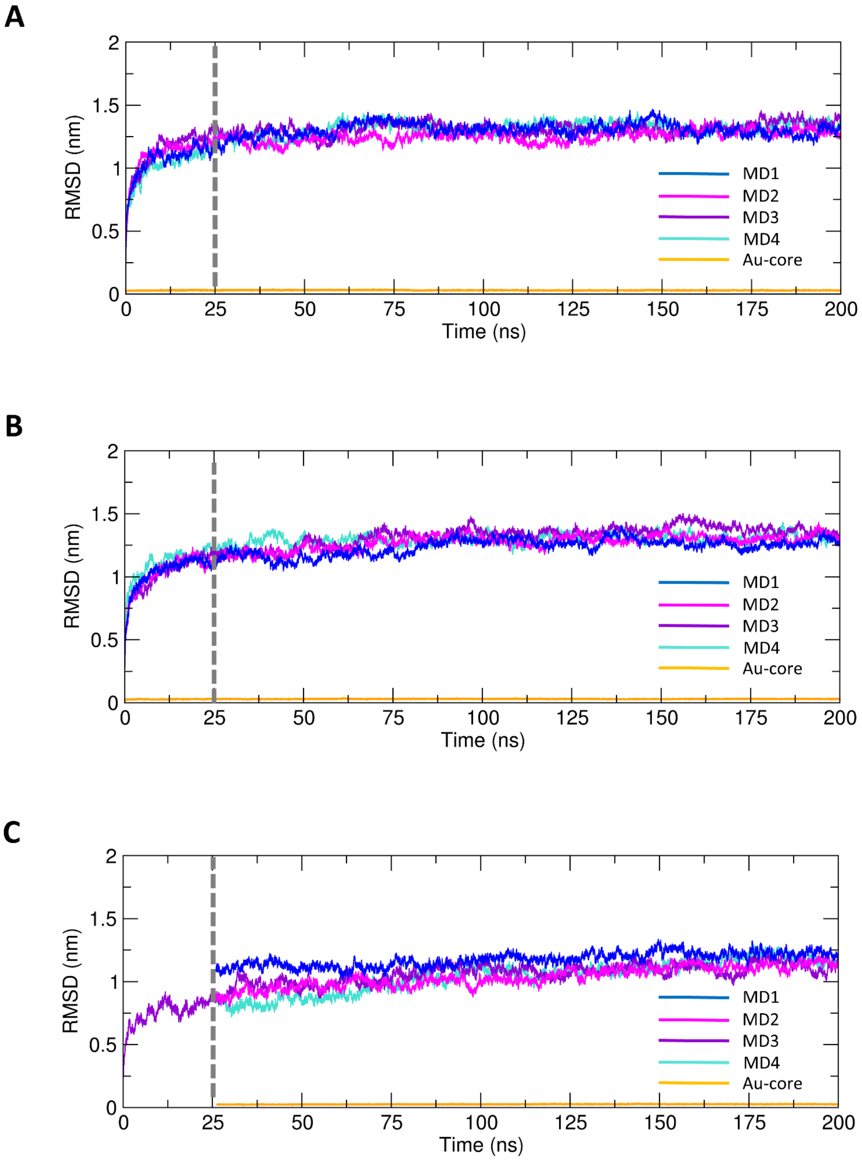

Figure S26. All-atom root mean square deviation (RMSD) for AuNP-**2** for which we run: (**A**) four 200 ns long simulations (MD1-MD4) at 25 °C without the presence of substrate; (**B**) four 200 ns long simulations (MD1-MD4) at 25 °C with the presence of substrate; (**C**) four 200 ns long simulations (MD1-MD4) at 40 °C with the presence of substrate. The RMSD of gold atoms is also reported (in orange), confirming that the gold core is stable during the simulation time. For all analysis discussed in the main text, we considered the data from 25 ns on as, after this time (gray dashed line), the systems are equilibrated.

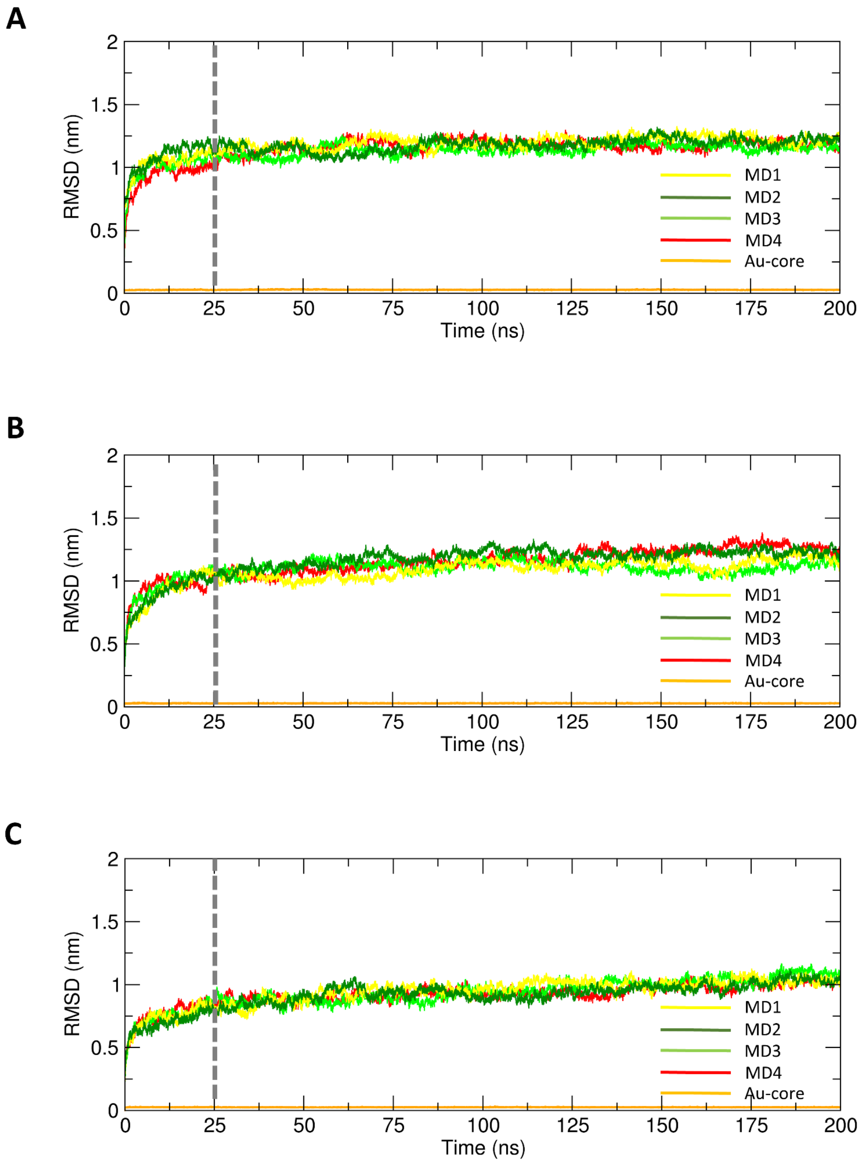

Figure S27. All-atom root mean square deviation (RMSD) for AuNP-**3** for which we run: (**A**) four 200 ns long simulations (MD1-MD4) at 25 °C without the presence of substrate; (**B**) four 200 ns long simulations (MD1-MD4) at 25 °C with the presence of substrate; (**C**) four 200 ns long simulations (MD1-MD4) at 40 °C with the presence of substrate. The RMSD of gold atoms is also reported (in orange), confirming that the gold core is stable during the simulation time. For all analysis discussed in the main text, we considered the data from 25 ns on as, after this time (gray dashed line), the systems are equilibrated.
